# Comprehensive genomic analysis of an indigenous *Pseudomonas pseudoalcaligenes* degrading phenolic compounds

**DOI:** 10.1038/s41598-019-49048-6

**Published:** 2019-09-04

**Authors:** Maryam Safari, Bagher Yakhchali, Vahid Shariati.J

**Affiliations:** 1 Department of Energy and Environmental Biotechnology, Institute of Industrial and Environmental Biotechnology, National Institute of Genetic Engineering and Biotechnology (NIGEB), Tehran, I. R. Iran; 2 Department of Biology, Faculty of Science, Nour Danesh Institute of Higher Education, Isfahan Province, Meymeh, Danesh Blvd, I. R Iran; 3 Department of Plant Molecular Biotechnology, Institute of Agricultural Biotechnology, National Institute of Genetic Engineering and Biotechnology (NIGEB), Tehran, I. R. Iran

**Keywords:** Bacterial genomics, Environmental sciences

## Abstract

Environmental contamination with aromatic compounds is a universal challenge. Aromatic-degrading microorganisms isolated from the same or similar polluted environments seem to be more suitable for bioremediation. Moreover, microorganisms adapted to contaminated environments are able to use toxic compounds as the sole sources of carbon and energy. An indigenous strain of *Pseudomonas*, isolated from the Mahshahr Petrochemical plant in the Khuzestan province, southwest of Iran, was studied genetically. It was characterized as a novel Gram-negative, aerobic, halotolerant, rod-shaped bacterium designated *Pseudomonas* YKJ, which was resistant to chloramphenicol and ampicillin. Genome of the strain was completely sequenced using Illumina technology to identify its genetic characteristics. MLST analysis revealed that the YKJ strain belongs to the genus *Pseudomonas* indicating the highest sequence similarity with *Pseudomonas pseudoalcaligenes strain* CECT 5344 (99% identity). Core- and pan-genome analysis indicated that *P*. *pseudoalcaligenes* contains 1,671 core and 3,935 unique genes for coding DNA sequences. The metabolic and degradation pathways for aromatic pollutants were investigated using the NCBI and KEGG databases. Genomic and experimental analyses showed that the YKJ strain is able to degrade certain aromatic compounds including bisphenol A, phenol, benzoate, styrene, xylene, benzene and chlorobenzene. Moreover, antibiotic resistance and chemotaxis properties of the YKJ strain were found to be controlled by two-component regulatory systems.

## Introduction

Environmental pollution is considered an important threat to the ecosystem^1^, influencing all organisms and human health extensively^1,2^. Traditionally, wastewaters and effluents are treated with chemical and physical methods. However, microbial decomposition of pollutants has also been widely investigated. Microbial degradation provides the possibility of complete mineralization of organic compounds^3^. Hence, bioremediation can be noted as a sort of medicine for the environment that can be applied to remedy polluted sites^1^. A large number of malicious organic compounds including halogenated aliphatics, pesticides and aromatic compounds such as benzene, xylene, toluene, phenolic compounds and their derivatives have been identified in the environment^4^. These contaminants are decomposed by a wide range of microorganisms including fungi, bacteria and yeasts^3,5^. Indigenous microorganisms from contaminated environments with the same type of pollutants are preferred for bioremediation of contaminants because they are adapted and can tolerate harsh environmental conditions and, therefore, may have more incentive to attack the pollutants^1^. Generally, a diverse set of catabolic enzymes involved in the conversion of aromatic compounds to Krebs cycle intermediates through the *ortho-* or *meta*-cleavage pathways, depend on bacterial genetic characteristics^6,7^.

*Pseudomonas* is a genus of Gram-negative, aerobic bacteria, containing several species that have been isolated from various environments, such as water and soil^8,9^. Some *Pseudomonas* species are capable of metabolizing chemical pollutants in the environment, and as a result, can be utilized for bioremediation^8^. In fact, *Pseudomonas alcaligenes*, *Pseudomonas mendocina*, *Pseuodomonas resinovorans* and *Pseudomonas pseudoalcaligenes* have been reported as suitable bioremediation agents^10–12^. In a recent research by Jahanshahi *et al*., the *Pseudomonas* YKJ strain was found to grow in presence of some aromatic pollutants as the only sources of carbon and energy^13^.

The genetic characterization of microorganisms isolated from the environment is momentous for conception of genetic mechanisms of adaptation processes, and regulatory and structural genes of catabolic pathways involved in environmental processes and application^14–17^. The advent of next generation sequencing (NGS) technologies, accompanied with the development of many new assembling methods and software programs, can provide access to the whole-genome sequence of a microorganism in a much shorter period of time and at lower costs than the traditional sanger sequencing method^18,19^. It is used for various purposes^16^ including evaluation of the presence or absence of a catabolic pathway in microorganisms^20^.

**Figure 1:**
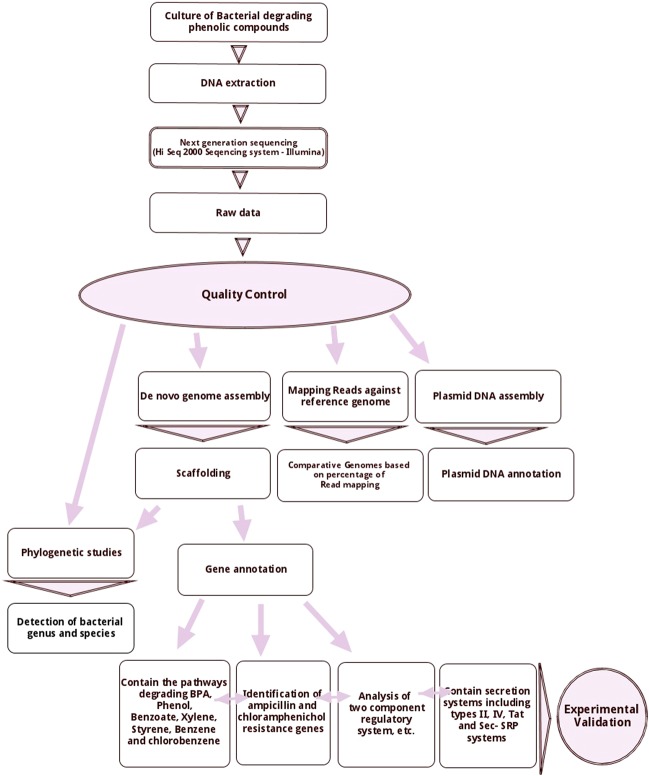
Workflow of the sequencing and analysis of the YKJ genome. It comprises sample preparation, next-generation sequencing (NGS) and data analyses including genome assembly, mapping reads, gene annotation, phylogenetic studies and identification and extraction of the plasmid DNA sequence. As shown in the flow chart, reads were mapped against the reference genome through filtering and trimming processes and were *de novo* assembled to determine a draft genome assembly. Subsequent analyses were carried out on the resulting YKJ genome.

In this study, the whole-genome sequence analysis of a previously isolated indigenous *pseudomonas* YKJ strain (GenBank accession no. KR229982) was carried out and compared with seven most similar strains using core- and pan-genome, and phylogenetic analyses to determine target genes and pathways related to the advantageous interactions between bacteria and their surrounding environment including genes involved in aromatic compound degradation pathways and regulatory systems in response to the chemical compounds (Fig. 1).

## Results

### DNA extraction

High quality genomic DNA of *Pseudomonas* YKJ strain, suitable for sequencing and molecular experiments, was extracted and used for NGS and amplification of the 16 s rRNA gene (Fig. S1–1).

### Genome sequencing and assembly

The qualified DNA was sequenced using the paired-end sequencing approach through a HiSeq 2000 sequencing Platform. A library of DNA fragments, 350 bp in size, was constructed and used for sequencing in both directions which resulted in 612,945,871 reads with an average length of 101 bp and G + C content of 63% (Table S2). The reads were filtered by removing low quality bases (≤Q20), repetitious contamination and trimming of 5′ ends to attain suitable conditions for analysis (Fig. S2). The cleaned reads were then analyzed using two approaches to assemble the genome. The Velvet assembler produced 592 contigs with an N50 size of 27295 bp and total size of 4.6 Mbp, using k-mers of 65. While, the genome sequence acquired through the SPAdes assembler revealed better results in assembly, consisting of 140 contigs (>500 bp) with lengths ranging from 687 to 307845 bp and an N50 size of 96476 bp, which ultimately resulted in a total length of 4,905,549 bp using k-mers of 65. Therefore, 140 scaffolds were considered as the genome of the strain (Table S3). Consequently, scaffolds created via the SPAdes assembler were annotated to detect target genes and pathways, which were then registered in the NCBI databank under the accession number PTLV00000000. In addition, BUSCO assessment showed that 99.8% of the genome was fully obtained.

### Phylogenetic analysis

**Figure 2:**
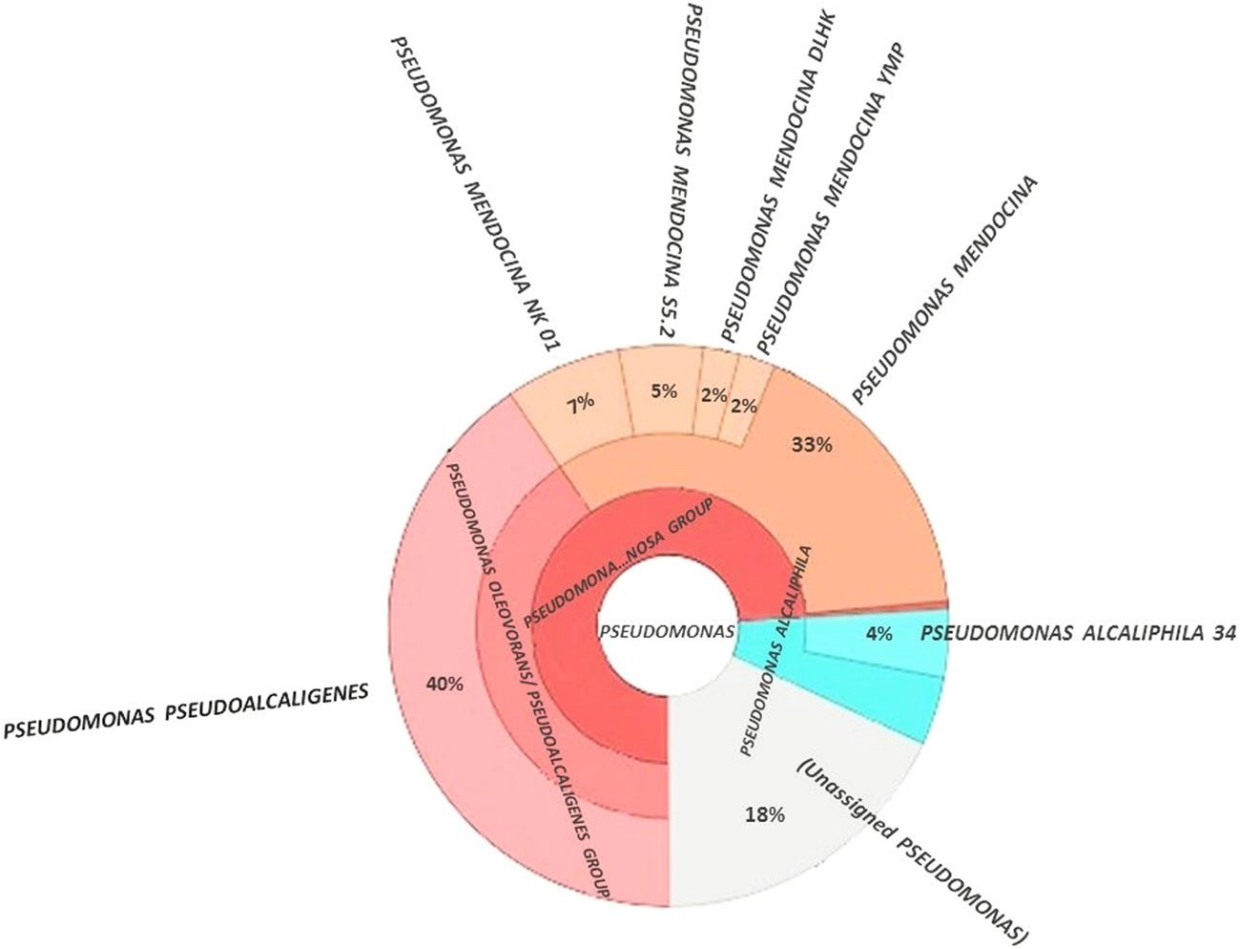
Taxonomic analysis of *Pseudomonas* sp. *YKJ* strain. Alignment of *YKJ* reads to universal marker genes in Phylosift, showed *pseudoalcaligenes* as the closest species.

**Figure 3:**
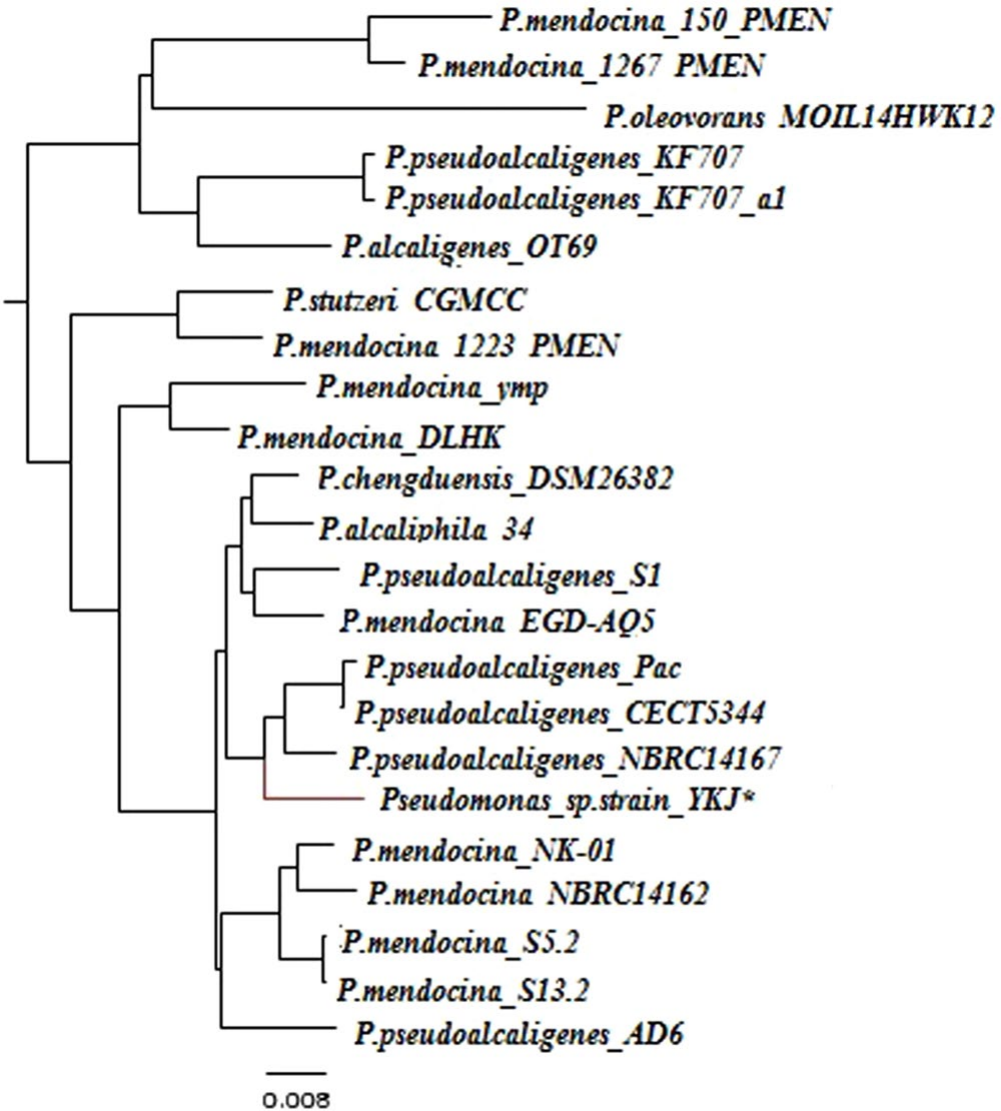
The CVTree of 23 bacteria. The tree is acquired with K = 6, based on the nucleotide sequence of all genes of the 22 strains related to the genus *Pseudomonas*.

Comparison of the 16S rRNA gene isolated from the YKJ draft scaffold against the NCBI 16S rRNA genes database showed that the YKJ strain belonged to the genus *Pseudomonas*, with *Chengduensis* being identified as the closest species (99%) (Table S4), which was consistent with the results reported by Jahanshahi *et al*.^13^. But, phylogenetic analysis of the individual *rpoD*, *rpoB*, and *gyrB* genes sequences as well as the 16S rRNA gene sequence in the PseudoMLSA database revealed that the strain *YKJ* belongs to the genus *Pseudomonas* and most closely resembles the *pseudoalcaligenes* species, strain *CECT5344* (Table S5). Consequently, the mapping and MLST results were confirmed by CVTree and PhyloSift analyses (Figs 2 and 3), which state that the target strain belongs to the genus and species of *pseudomonas pseudoalcaligenes*.

### *Pseudomonas pseudoalcaligenes YKJ* genome features and whole-genome comparison

The percentage of matching reads to the 7 relevant genomes was investigated to find the best reference genome (Fig. S3). Ultimately, the *P*. *pseudoalcaligenes* strain CECT5344 (GenBank: GCA_000297075.2) was selected as the closest strain with alignments of over 63% of the reads (Table S9).

**Figure 4:**
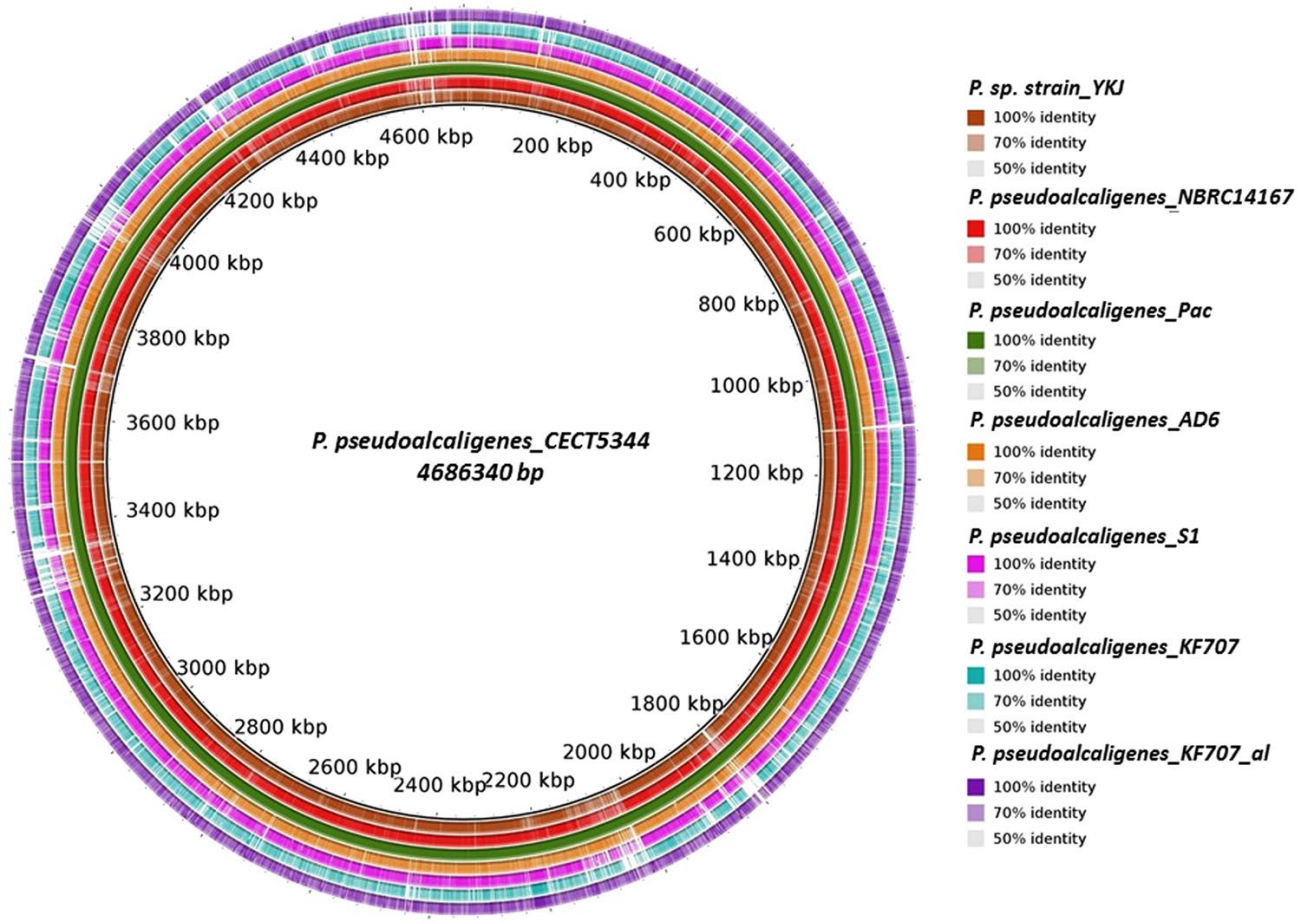
Genome comparisons of *Pseudomonas pseudoalcaligenes* YKJ and other *P*. *pseudoalcaligenes* strains against strain CECT5344 as the reference genome created by BRIG v0.95, the circular graph demonstrates the whole genome comparison of strain CECT5344 with the other seven closely-related genomes of *P*. *pseudoalcaligenes*. The inner black circle reveals the complete reference genome (strain CECT5344) and the intensity of each color indicates the similarity of that strain with the reference genome.

**Figure 5:**
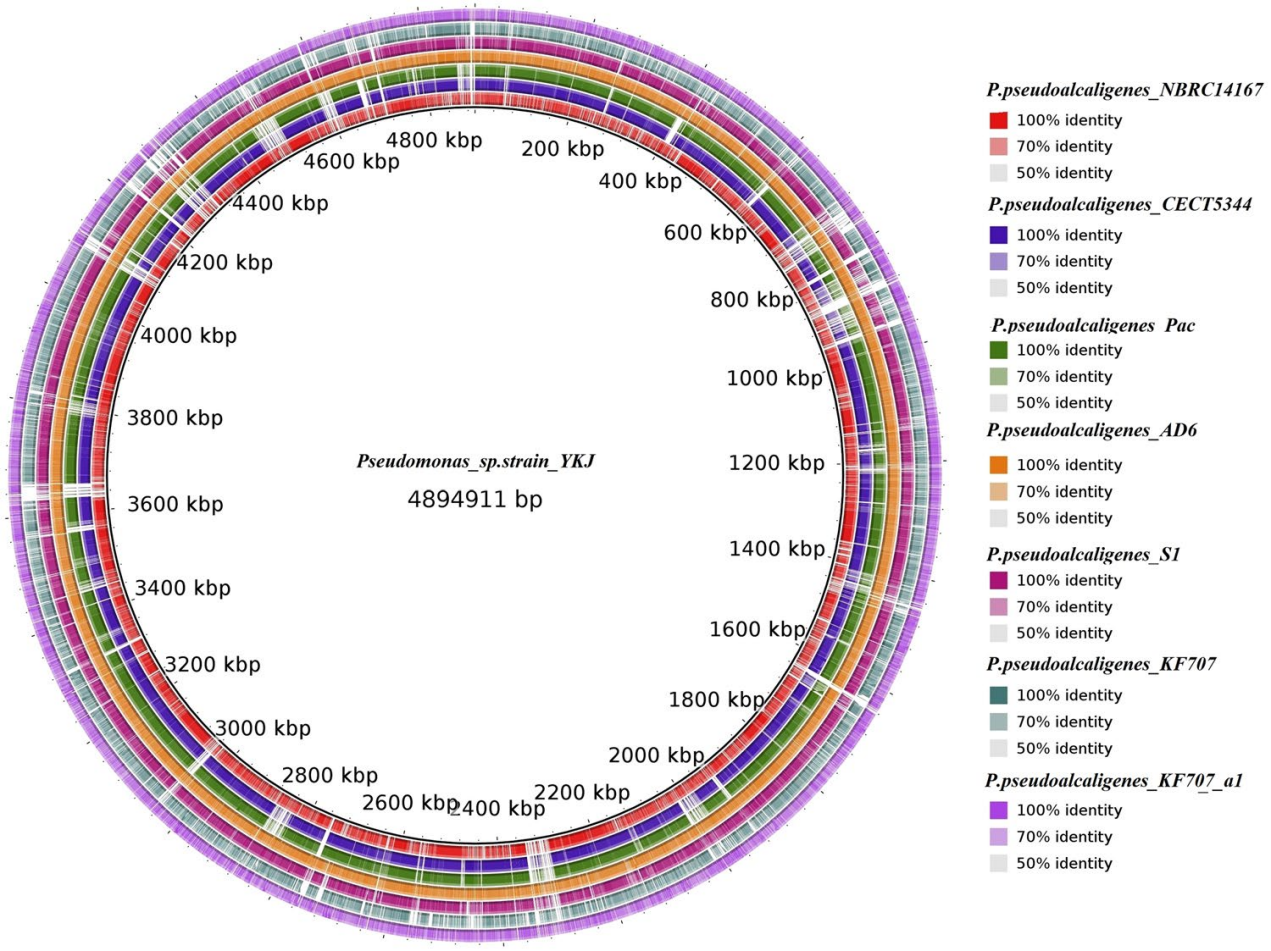
Genome comparisons of other *P*. *pseudoalcaligenes* strains against YKJ strain scaffold. The inner black circle indicates the whole genome of the YKJ strain as the reference genome and the intensity of each color indicates the similarity of that strain with YKJ strain.

The draft genome assembly of *P*. *pseudoalcaligenes* YKJ includes a single circular chromosome of 4.9 Mbp with an average GC content of 63%. In total, 4583 genes were predicted in this genome, encoding 4565 putative coding sequences (CDS). Moreover, 48 tRNA genes and 3 rRNA genes were predicted in the chromosome sequence. The complete characteristics of *P*. *pseudoalcaligenes* YKJ and seven closely-related genomes are presented in Supplemental Table S7, and Figs 4 and 5. Accordingly, based on genomic sequence and other features including the genome size, total number of genes and total predicted CDS, *P*. *pseudoalcaligenes* YKJ is considered as the strain most similar to *P*. *pseudoalcaligenes* CECT5344.

**Figure 6:**
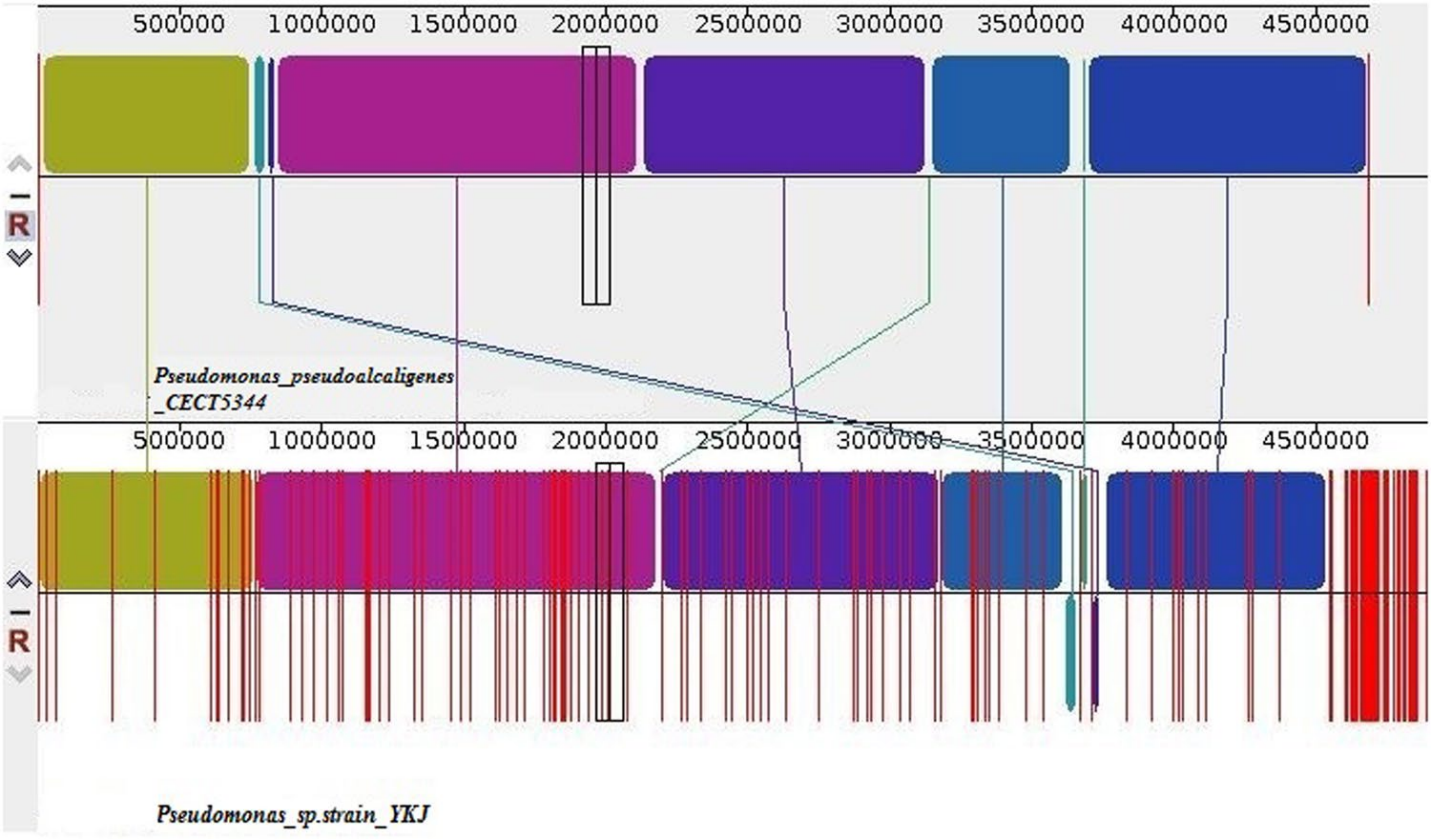
Genome alignment illustrating syntenic blocks between the *P*. *pseudoalcaligenes* strain CECT5344 (upside) and the YKJ genome (downward).

Using Mauve analysis, we found 9 collinear blocks between concatenated chromosomes of two strains (Fig. 6), showing several inversion and rearrangement structures. In addition, the chromosomal alignments showed existence of large segments with high similarity. Moreover, a region of approximately 2 Mb between contigs 4 to 8 in the YKJ chromosomal scaffolds displayed inversion, revealing different reciprocal relationships to the reference sequence. It was also shown that the YKJ chromosomal scaffolds have an insertion region between contigs 5 to 7.

### Pan- and core-genome analysis of *P*. *pseudoalcaligenes* YKJ

**Figure 7:**
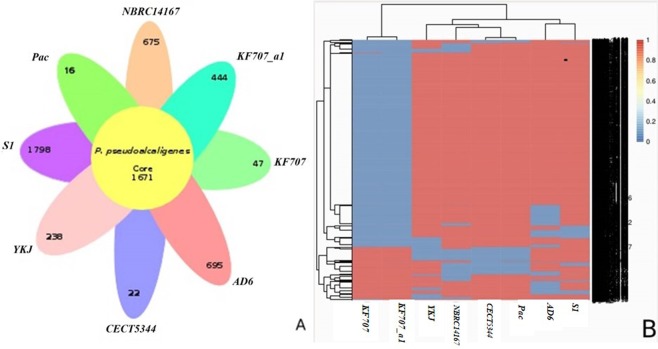
Plot of the core-and pan-genome and Venn diagram for the core-genome and strain-unique CDS of the *P*. *pseudoalcaligenes* strains. (**A**) The number of accessory CDS for each strain of the *P*. *pseudoalcaligenes* pan-genome analysis. (**B**) Pheatmap of *P*. *pseudoalcaligenes* accessory (shell) genes.

A pan-genome analysis for YKJ and the 7 sequenced *P*. *pseudoalcaligenes* strains was performed using the Roary tool based on comparison of the translated CDS set. The genome of 8 *P*. *pseudoalcaligenes* strains comprised a total of 13,431 genes consisting of 1,671 genes in the core-genome and 11,760 genes in the accessory genome, including 7,443 shell genes (moderately common in the pan-genome) and 4,317 cloud genes (present in very few analyzed genomes). With regard to the accessory genome, 3,935 genes are present in the unique genomes, indicating the existence of specific genes among the *P*. *pseudoalcaligenes* strains. The lowest numbers of unique genes belonged to the Pac, CECT5344 and KF707 strains, with 16, 22 and 47 genes, respectively. Strains S1, NBRC14167, AD6 and KF707_a1 contained the highest numbers of specific genes as well (Fig. 7). The target genome (YKJ strain) contained 238 unique genes, 97 of which have been predicted to encode hypothetical proteins (Table S8).

The ability of the YKJ strain to use aromatic compounds (pollutants) as carbon and energy sources was also analyzed. This strain contains all the genes that contribute to the ortho-cleavage pathway. Furthermore, it contains the full complement of genes involved in the meta-cleavage pathway. The *tfdS* and *glpF* genes were also identified in the YKJ strain, which act as transcriptional activator for the *bauABCD* operon^21^ and glycerol-3-phosphate regulon repressor^22^, respectively. Moreover, 34 known genes belonging to transcription regulators, hydrolases, the toxin-antitoxin system and transferases were also identified. (Table S8).

### Relatedness of *Pseudomonas pseudoalcaligenes* YKJ based on core- and pan-genome analysis

**Figure 8:**
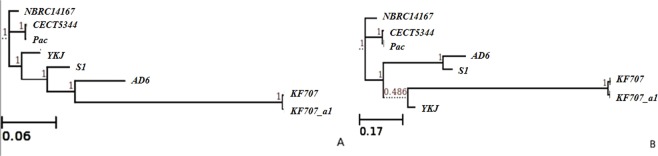
Phylogeny of the *P*. *pseudoalcaligenes* strains based on core gene alignments (**A**) and accessory genes analysis (**B**).

The phylogenetic relationship of *P*. *pseudoalcaligenes* YKJ and the other seven *P*. *pseudoalcaligenes* strains were revealed based on the core- and pan-genome analysis. As shown in Fig. 8B, *P*. *pseudoalcaligenes* YKJ is clustered in a separate clade based on the pan-genome analysis, which is genetically close to the KF707_a1 and KF707 strains. A phylogenetic tree was also created using the amino acid sequences of 1671 genes in the core-genome, which showed the clustering of the YKJ strain in a separate clade, with a close phylogenetic relationship between the YKJ and Pac, NBRC14167 and CECT5344 strains. Furthermore, this analysis also revealed a distant phylogenetic relationship between NBRC14167 and KF707_a1 and KF707 strains that were placed in the same clade (Fig. 8A).

### Pathway analysis

Annotation of the *P*. *pseudoalcaligenes* YKJ genome led to prediction of approximate biochemical, catabolic and biosynthetic pathways, as shown in Supplemental Fig. S4. Classification of the metabolism groups also revealed genes involved in each group (Fig. S5). Genome annotation identified key genes associated with the metabolism of aromatic compounds, II and IV secretion systems, antibiotic resistance, and carbon metabolism, etc. (Tables S10–S14).

### Aromatic compound degradation pathways

Analysis of the YKJ strain genomic data identified approximately 117 candidate genes probably involved in the degradation of benzene, chlorobenzene, xylene, styrene, benzoic acid, phenol and bisphenol A. The data also revealed S-hydroxymethyl dehydrogenase and alcohol dehydrogenase genes, as well as ammonia monooxygenase and flavin-dependent monooxygenase genes, which contribute to the conversion of aromatic compounds to catechol (Table S10). However, the YKJ genome also possesses both catechol 1, 2-dioxygenase and catechol 2, 3-dioxygenase genes which are involved in the degradation of catechol in the ortho- and meta-cleavage pathways, respectively (Table S9). As reported in Supplemental Table S9, the YKJ strain genome contains all the genes implicated in the *ortho*- and *meta*-cleavage pathways. In addition, we could not find a complete group of genes related to toluene degradation.

### Secretion systems

**Figure 9:**
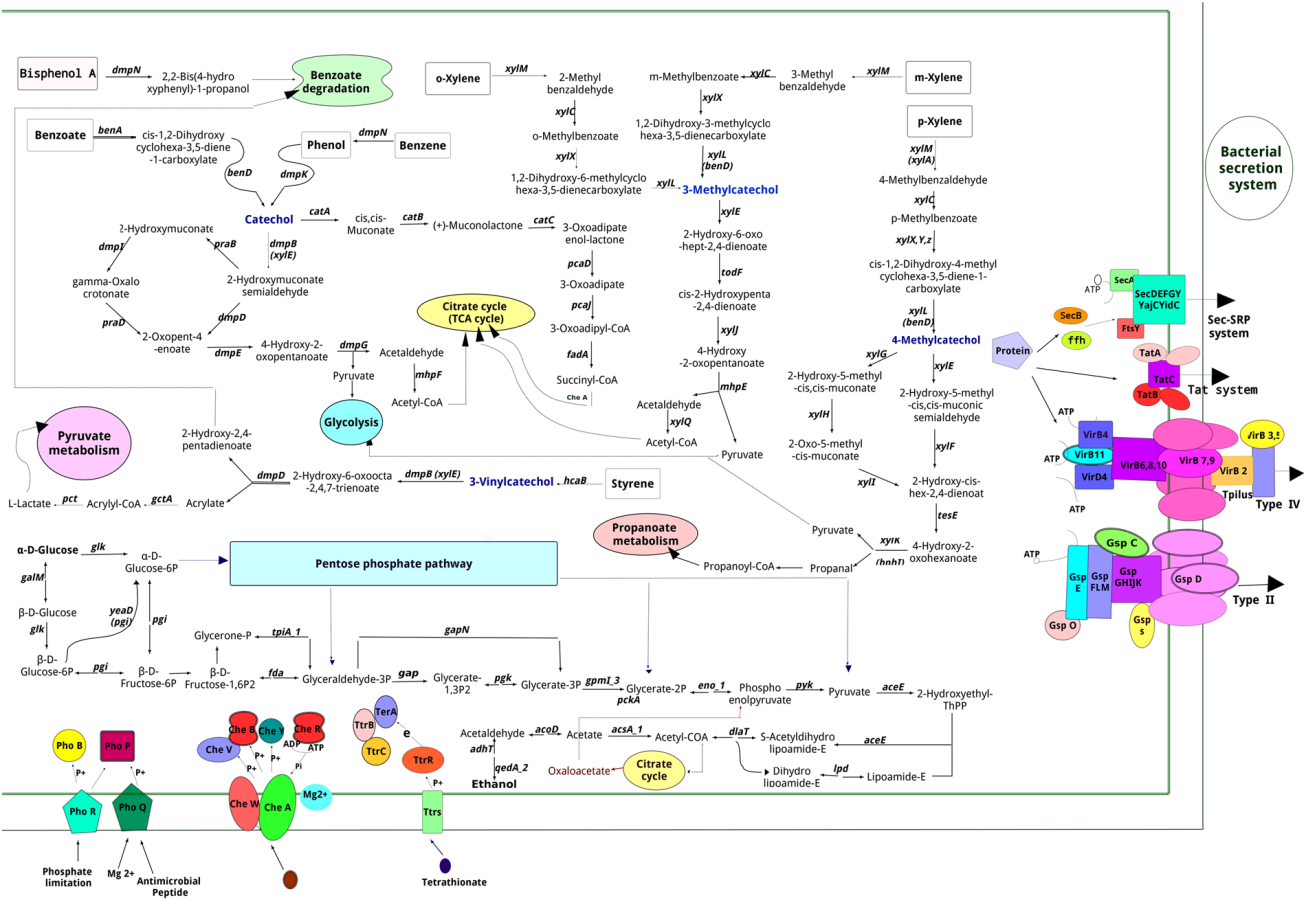
Schematic overview of metabolic pathways of *P*. *pseudoalcaligenes* YKJ. The described pathways were found on the basis of genomic data, indicating the strain contains genes related to the ability of degrading aromatic compounds. A complete carbohydrate metabolic route including glycolysis, gluconeogenesis, Krebs cycle, the pentose phosphate pathway (PPP) and pyruvate metabolism are also present in the YKJ genome. The latter comprises the genes contributing to the secretion systems involving T4SS, T2SS, Tat and Sec-SRP systems. Several genes related to two-component regulatory systems are also available in the YKJ strain.

Two outer-membrane protein secretion systems consisting of types II and IV, as well as the Tat (twin arginine translocation) and Sec-SRP (general secretory pathway) systems were identified in the YKJ genome. All genes encoding protein subunits involved in the T4SS, T2SS and Sec-SRP systems were detected in the YKJ genome (Table S11, Fig. 9), whereas only the *tolC* gene related to the type I secretion systems (TISS) was identified in this strain.

### Quorum sensing

We found 94 genes related to two-component regulatory systems in the YKJ genome, such as the PhoP-PhoQ, PhoR-PhoB, NtrB-NtrC and CheA-CheYBV systems (Table S12, Fig. 9). The YKJ genome also contains 41 genes involved in the quorum sensing process, which may act directly through these genes or indirectly through cellular metabolism (Table S13). Of those genes, *qseC* and *qseB* genes are the membrane sensor kinase and response regulator of the two-component QseB-QseC signaling system, respectively. The QseC enzyme responds to bacterial signals by phosphorylating the QseB response regulator, leading to its activation and positive regulation of flagella and motility genes^23,24^. In addition, the *qseC* and qseB genes are components of the quorum-sensing system, which could mean that the quorum-sensing and two-component systems interact with each other^25^.

### Antibiotic resistance in the YKJ strain

By interpreting the genomic data from the YKJ strain, we found approximately 31 candidate genes implicated in antibiotic resistance pathways (Table S14). These included genes that encode beta-lactamases and chloramphenicol acetyltransferases, which allow bacteria to grow in the presence of ampicillin and chloramphenicol antibiotics, respectively^26^.

### YKJ plasmid DNA detection and assembly

Plasmid sequence was separately acquired in 39 scaffolds (≥500 bp), which resulted in a total length of 276,024 bp with an N50 size of 12,722 bp. Using the PlasmidFinder server, the incompatibility (Inc) group of the plasmid from the YKJ strain (pYKJ) was identified as the Incp1 (beta) group. pR751 was also recognized as the closest plasmid to pYKJ (Table S15).

Comparison of pYKJ nucleotide sequence with eight other plasmids, namely pRms149, pTOM9, pKLC102, pRA2, pDK1, pWW53, pPME5 and pR751, revealed their similarities and differences (Figs S6 and S7).

### *YKJ* plasmid DNA annotation

Analysis of the pYKJ scaffold in Addgene server indicated the presence of *Pme*l and *Swa*l restriction enzymes sites, as well as ORFs (Fig. S8). Moreover, annotation of the pYKJ scaffold recognized all genes encoding type IV secretion system proteins associated with conjugative plasmid transfer (Table S16). As presented in the Supplemental Table S17, antibiotic resistance genes are not present on pYKJ and several genes are of genomic origin. In total, 262 protein CDS and eight tRNA-coding genes were identified in the pYKJ plasmid.

### Experimental analysis of aromatic compound degradation pathways

Performance of the degradation pathways found in the YKJ strain through sequence analysis was investigated by growth in mineral salts medium supplemented with different aromatic compounds as sole sources of carbon and energy.

The growth of bacterial cultures (OD_600_) revealed that the YKJ strain is able to grow in the presence of bisphenol A, phenol, styrene, xylene, benzene, chlorobenzene and benzoic acid. Furthermore, using various concentrations of each pollutant indicated that the YKJ strain is capable of growing at specific concentrations (Fig. S9). Accordingly, we identified the relevant concentrations (Table S18) of aromatic compounds as the sole sources of carbon and energy, which enabled maximum growth of the YKJ strain. Hence, this strain is more capable of decomposing and consuming bisphenol A, phenol and styrene as the only sources of carbon and energy than xylene, benzene, chlorobenzene and benzoic acid (Fig. S10).

### Antibiotic resistance in the YKJ strain

Antibiotic resistance was investigated via cultivation of the strain on LB agar medium containing an antibiotic. According to the results, the YKJ strain is resistant to ampicillin and chloramphenicol while the growth intensity of this strain was higher on the LB agar medium supplemented with chloramphenicol (Table S19). The results confirmed the function of the predicted genes encoding β-lactamase and chloramphenicol acetyltransferase.

The antibiotic resistance was also investigated after transfer of pYKJ into *E*. *coli* DH5α, which showed no growth of transformants on ampicillin- and chloramphenicol-containing media. The presence of pYKJ in the five antibiotic sensitive transformants was verified by plasmid extraction and agarose gel electrophoresis (Fig. S11).

## Discussion

In this study, we carried out *in silico*, genetic and biochemical characterization of an indigenous *Pseudomonas* YKJ strain through whole-genome sequencing. The genomic data was confirmed and supported by experimental tests. The comparative genomic analysis of *P*. *pseudoalcaligenes* YKJ and seven previously sequenced *P*. *pseudoalcaligenes* strains revealed high similarity amongst them, particularly the Pac, CECT5344 and NBRC14167 strains. According to the pan-genome analysis, the YKJ strain was genetically clustered in a separate clade close to the KF707_a1 and KF707 strains, which are clustered in the same clade (Fig. 8B). Moreover, a phylogenetic tree based on the amino acid sequences of 1671 genes of the core-genome demonstrated low diversity between the studied strains, revealing their evolution from a common ancestry (Fig. 8A).

There are two main strategies to decompose aromatics, which are dependent on the absence or presence of oxygen^27^. Under aerobic conditions, biodegradation is carried out via the *ortho*- or *meta-*cleavage pathways^28^. The main genes involved in the meta-cleavage pathway include the *dmpB*, *dmpC* and *dmpD* genes encoding catechol 2,3-dioxygenase, 2-hydroxymuconic semi-aldehyde dehydrogenase and 2-hydroxymuconic semi-aldehyde hydrolase, respectively^29^. In addition, the *catRBCA* gene cluster is involved in the *ortho*-cleavage pathway that converts catechols to 3-oxoadipate intermediates^30^. Here, our studies revealed that the YKJ strain contains the *catRBCA* gene cluster and all the other genes associated with the meta-cleavage pathway (Table S9). Pan-genome analysis of eight strains revealed the presence of the complete *catRBCA* gene cluster in only five strains, namely, strains YKJ, AD6, KF707_a1, KF707 and S1 (Table S8). Furthermore, the strain YKJ contains all the genes that play a part in the meta-cleavage pathway, whereas the *dmpB*, *dmpC* and *dmpD* genes are only present in the S1 strain genome.

The YKJ strain contains enzyme-coding genes involved in pathways that degrade certain aromatic compounds, namely phenol, bisphenol A, benzene, chlorobenzene, styrene, xylene and benzoic acid. Phenol degradation is reported in many *Pseudomonas* strains such as *Pseudomonas* sp. CF600, which grows efficiently on phenol using the meta-cleavage pathway encoded by the p*VI150* plasmid carrying the *dmpKBCIHEGF* or *dmpKBDEGF* genes organized into operons^7^. The YKJ strain contains the complete *dmpKBDCIHEGF* genes (Table S10) in its genome and can grow on phenol as a preferred sole carbon and energy source (Figs S9b and S10).

Although, several pathways have been presented for aerobic degradation of bisphenol A, there have been few studies investigating genes and enzymes related to the BPA degradation pathways. The first BPA-degrading pathway was characterized in the *Sphingomonas* sp. strain MV1^31^. A metabolic pathway for bisphenol A metabolism by Gram-negative bacteria^32^ has also been reported. In these studies, a cytochrome P450 monoxygenase in S*phingomonas*, an ammonia monooxygenase (AMO) in *Nitrosomonas europaea* and an extracellular laccase in *Pseudomonas* have been also implicated in BPA degradation^31,33^. Thus, enzymes involved in BPA degradation have not yet been fully characterized. Based on the genomic analysis, the *YKJ* strain has been found to contain monoaxygenase-coding genes such as AMO. In addition, experimental results revealed that the *YKJ* strain is capable of growing on the medium containing BPA as the sole carbon and energy source (Fig. S9a). Our previous study^13^ showed that 4-hydroxyacetophenone, 4-hydroxybenzaldehyde and 4-hydroxybenzoic acid were all produced during BPA degradation by this strain. These BPA metabolites have been recorded in the BPA degradation pathway in the KEGG database (PATHWAY: map00363). Therefore, the genome of the YKJ strain contains genes encoding BPA degradation pathway enzymes, and the strain prefers to use bisphenol A (0.3 g/l) in addition to phenol (1 mM) and styrene (0.1% v/v) (Fig. S10). Since the amounts and activities of the enzymes contributing to the BPA degrading pathway are remarkably diverse in various bacterial strains^31^, it is essential to characterize the intermediates formed during the enzymatic degradation of BPA. Therefore, the new genes and enzymes involved in BPA decomposition need to be further investigated.

In a study on the degradation of aromatic compounds by the strain, *Pseudomonas putida* KT2440, benzoate is metabolized through conversion into catechol and then to Krebs cycle intermediates^34^ (*ortho*-cleavage) and can thus be used by the bacterium as a sole source of carbon and energy^35^. The analysis of YKJ strain genome indicates the presence of all *dmpKBDCIHEGF* genes involved in the *ortho*-cleavage pathways (Table S10), therefore, it should be capable of degrading benzoate. This was confirmed by the ability of the strain to grow in medium consisting of benzoate as the sole carbon and energy source (Fig. S9e). Degradation of chlorine- or methyl-substituted aromatic compounds derivatives, such as chlorobenzene^36^ is performed via two-enzymatic systems that include dioxygenases (upper pathway, attack the aromatic ring) and monooxygenase (lower pathway, attack the methyl or ethyl substituents of the aromatic ring)^37^. The *xylUWCMABN* operon (upper pathway) oxidizes methylbenzenes to methyl benzoates and the meta-operon (lower pathway, *xylXYZLTEGFJQKII-I* genes) converts methylbenzoates to pyruvate, acetaldehyde and acetate through the (methyl) pyrocatechols. Our study revealed that the *xyl XYZLEGFJQKI* genes are present in the YKJ genome (Table S10). Thus, it is predicted that this strain converts *o-* and *m-*xylene to acetaldehyde, and *p*-xylene to propanol through the lower pathway (meta-operon). Notably, the *xylM* and *xylC* genes which oxidize *o-*, *m*- and *p*-xylene to *p*-methylbenzaldehyde via the upper pathway^38^ are absent in the YKJ genome. Hence, this strain cannot decompose xylene by the upper pathway while growing on the medium containing xylene as the only carbon source (Fig. S9d), thereby confirming the possible ability of the strain to degrade xylene through the lower pathway or a new pathway that should be further investigated.

Benzene-degrading bacteria contain mono- or dioxygenase enzymes, which convert benzene to phenol or *cis*-benzodiazolidine and then to catechol, the ring structure of which is ultimately cleaved in the *ortho* or meta position^39^. The chlorobenzene degrading pathway in *Pseudomonas* P51^40^ is encoded by a plasmid-located transposon, Tn5280. Homology comparisons revealed that these genes are most closely related to the *todC1C2BAD* genes (toluene degradation) and *bnzABCD* genes (benzene degradation), and distantly to genes involved in biphenyl and benzoate degradation. Similar to enzymes in the toluene degradation route, chlorobenzene dioxygenase and *cis*-chlorobenzene dihydrodiol dehydrogenase were able to oxidize 1, 2-dichlorobenzene, biphenyl and toluene, but not benzoic acid, which strongly suggest that these two enzymes originated from a pathway degrading toluene or benzene, likely via horizontal gene transfer^40^. The YKJ genome encompasses the genes encoding catechol 1, 2-dioxygenase, chloromuconate cycloisomerase and dienelactone hydrolase enzymes, which convert 3-chlorocatechol to maleylacetate. However the genome does not contain the *tcbA* and *tcbB* genes, which oxidize chlorobenzene to 3-chlorocatechol^41^, and the genes encoding benzene 1,2-dioxygenase and *cis*-1,2-dihydrobenzene-1,2-diol dehydrogenase, which convert benzene to catechol^42^ (Table S10). Additionally, we found complete gene sets contributing to phenol degradation, such as the *dmpK* gene, encoding phenol hydroxylase, which converts benzene to phenol (PATHWAY: map00362). Therefore, it seems that this strain is capable of converting benzene to phenol in its degradation route. Furthermore, styrene dioxygenase and *cis-*1,2-dihydrobenzene-1,2-diol dehydrogenase enzymes responsible for the oxidation of styrene to 3-Vinylcatechol^43^, were absent, while the *xylE*, *xylF*, *catA*, *gctA*, *gctB*, *feaB* and *pct* genes were present in the YKJ genome (Table S10). Experimental data also revealed that this strain is capable of growth on chlorobenzene, benzene and styrene as the only sources of carbon and energy (Fig. S9c,f,g). Due to considerable variety and specific functional properties, various monooxygenase enzymes are able to degrade aromatic compounds by aromatic ring cleavage. For instance, a phenol hydroxylase belonging to the flavin-dependent monooxygenase family has been identified in *Bacillus thermoglucosidasius* A7, which catalyzes *ortho*-hydroxylation of phenol to catechol^44^. The vinyl side chain oxidation is catalyzed via a FAD-dependent styrene monooxygenase, leading to production of styrene epoxide^45^. Other microorganisms, such as *Pseudomonas Pickettii* PKO1^46^, also use flavoprotein monooxygenases to hydrolyze phenol. Costa *et al*. have stated^47^ that mesophilic microorganisms are aerobically capable of catabolizing the xenobiotic aromatic compounds by adding a hydroxyl group to the ring. Accordingly, flavoprotein monooxygenases (FPMOs) are often employed to perform this primary catabolic phase. The resulting catechol can then be cleaved by metal ion-dependent dioxygenase enzymes, resulting in the further cleavage of products to generate energy early in the metabolic pathways^48^. So, these studies confirm that flavin-containing enzymes are involved in the microbial decomposition of aromatic compounds. The study of the YKJ genome revealed genes encoding flavin-dependent monooxygenase enzymes including flavin monooxygenase and ammonia monooxygenase, as well as aromatic ring-cleavage dioxygenase enzymes (Table S10). Therefore, it can be concluded that the YKJ strain probably catabolizes the primary steps of benzene, chlorobenzene, xylene and styrene degradation through flavin-dependent monooxygenases, resulting in the formation of the corresponding catechols. The catechols are then degraded through the *ortho-* or *meta*-cleavage pathway, and eventually converted to the Krebs cycle intermediates. Nevertheless, the YKJ strain tends to consume phenol, bisphenol A and styrene more than benzene, chlorobenzene, xylene and benzoic acid. Existence of the above discussed genes encoding aromatic compound-degrading enzymes in the genome of our strain (instead of the plasmid or transposon) makes it suitable for application in bioremediation of aromatic compounds.

During the primary characterization of the YKJ strain, the 16S rRNA gene study revealed that it belongs to the *Pseudomonas chengduensis*^13^ species. The mentioned 16S rRNA gene was aligned against the final scaffolds, which did not completely overlap with sequences existing in the scaffolds. As mentioned in some studies, bacterial classification based on the 16S rRNA gene has not yet reached its complete potential, because many microbial taxonomic groups have not been detected yet^49^. Therefore, the use of other methods was required to accurately determine the genus and species of the YKJ strain. MLST, which is a procedure for typing of multiple loci that distinguishes microbial isolates through the comparison of the housekeeping gene sequences, such as *rpoD*, *rpoB* and *gyrB*, at the genus and species level^50^, has been used for phylogenetic relationship studies^51^. Thus, analysis of housekeeping genes is the preferred method in demonstrating the phylogenetic relationships among microbial genera and species^52^. The MLST studies based on *rpoD*, *rpoB* and *gyrB* gene sequences indicated that the YKJ strain belongs to the genus *Pseudomonas* and species *pseudoalcaligenes*. Some new phylogenetic studies have been conducted on the basis of all proteins or genes sequences^53^. Therefore, the phylogenetic relevance of the YKJ strain and 22 other strains were also investigated by aligning protein sequences of all strains. Consequently, the YKJ strain belongs to the genus *Pseudomonas* and species *pseudoalcaligenes* (Fig. 3). Phylogenetic studies at the reads level also confirmed these results (Figs 2, S3). In conclusion, the YKJ strain is considered as a *pseudoalcaligenes* species belonging to the genus *Pseudomonas*.

Resistance to ampicillin and chloramphenicol in Gram-positive and Gram-negative bacteria is dependent on the presence of β-lactamases and chloramphenicol acetyltransferases, which hydrolyze ampicillin and chloramphenicol, respectively^26^. The YKJ strain contains nearly 31 genes, mainly *catB* and *ampC* genes, involved in antibiotic resistance (Table S14). Hence, we predicted that the YKJ strain is resistant to these antibiotics. Hence, the antibiotic resistance of the YKJ strain was investigated by cultivation of the bacterium on medium containing ampicillin, tetracycline, kanamycin, rifampicin and chloramphenicol. As reported in Supplemental Table S19, the YKJ strain is resistant to ampicillin and chloramphenicol. Genome-encoded antibiotic resistance lessens the possibility of the horizontal transfer of the antibiotic resistance genes, which again makes this strain more suitable for environmental application.

Type IV secretion systems are diverse multi-functional systems with similar function in Gram-positive and Gram-negative bacteria^54^, which are usually composed of 12 subunits that are connected to an ATPase system in the membrane. Our study revealed that the YKJ strain contains all the genes (Table S11) encoding the protein subunits related to the VirB/D type IV secretion system^55^. The YKJ strain also comprises 12 *gsp* genes encoding the type II secretion system components (Table S11). Moreover, the *gsp* gene identification codes were found based on the KEGG Orthology (KO) database.

The general Sec-SR and Tat secretion pathways are frequently used by bacterial cells to transfer proteins across the cytoplasmic membrane. As presented in the Supplemental Table S11, the YKJ strain contains a family of *sec* genes that include the *secY* and *secE* genes encoding integral membrane proteins, and a *sec A* gene encoding an ATPase protein that acts as a molecular motor^56^. It also contains the SecB-coding gene, which functions as a chaperone to guide secretory proteins to the Sec-translocase^57^. We also found *tatA*, *tatB* and *tatC* genes in the YKJ genome (Table S11).

Two-component regulatory systems are widely present in bacteria that assist them in adapting to environmental conditions^58^. These regulatory systems monitor the internal and external signals and regulate various cellular processes, such as antibiotic resistance, metabolite utilization, reaction to environmental stress and sporulation^59^. In this study, we found 93 genes in the YKJ genome which are probably involved in the two-component regulatory pathways (Table S12). Among them, we can point to two important systems including CheA-CheYBV and PhoP-PhoQ. As reported by Gooderham, the pathogenicity and antibiotic resistance of *Pseudomonas aeruginosa* is controlled by the PhoP-PhoQ system which regulates resistance to aminoglycosides, polymyxin B and antimicrobial peptides^60^. On the other hand, the role of the CheA-CheYBV system in response to a variety of chemical and physical stimuli from the environment has been indicated in *Escherichia coli* and *Salmonella typhimurium*^61^. Since the YKJ strain is resistant to some antibiotics and is capable of growing on certain aromatic compounds as sole carbon and energy sources, by acquiring these two systems, it might be capable of responding to chemical stimuli controlled by the CheA-CheYBV system and antibiotic resistance monitored by the PhoR-PhoQ system.

Although, the antibiotic resistance genes such as those coding for chloramphenicol acetyltransferase and β-lactamase are generally located on episomal DNAs^26^, these genes are detected on the chromosomal DNA in the YKJ strain (Table S17). Hence, the YKJ plasmid comprises no antibiotic resistance genes which has been confirmed through the transfer of the p*YKJ* plasmid into *E*.*coli* DH5α. There are several studies indicating the presence of genes involved in aromatic compound degradation pathways on plasmids^7,40^. Our results showed that as well as antibiotic resistance genes, the YKJ chromosomal DNA contains all the relevant genes involved in the aromatic compound degradation pathways. Hence chromosol location of such genes can be considered a positive feature and an advantage for the environmental application of this strain, because the possibility of transferring and losing these genes ought to be relatively low. Moreover, these two features, aromatic compound degradation and antibiotic resistance, were preserved in the studied experimental population of the strain over many consecutive generations. In addition, many of the reported genes in Supplemental Table S17 are related to genomic DNA rather than plasmid.

## Conclusion

In this study, we sequenced and analyzed the whole genome of an indigenous *Pseudomonas* sp., namely YKJ that was able to degrade phenolic compounds. Phylogenetic studies based on the housekeeping and all gene sequences indicated that the YKJ strain belongs to *Pseudomonas pseudoalcaligenes*. The whole-genome comparison of *P*. *pseudoalcaligenes* YKJ and seven closely related *pseudoalcaligenes* strains indicated 3935 and 1671 CDS as accessory and core-conserved genes, respectively. Investigation of its metabolic features revealed the YKJ’s ability to degrade several aromatic compounds either through the *meta-* or *ortho*-cleavage pathways. This strain contains antibiotic resistance genes including *ampC* and *catB* that code for the β-lactamase and Chloramphenicol acetyltransferase enzymes, respectively. Furthermore, the YKJ genome comprises a set of genes encoding the T4SS, T2SS, Tat and Sec-SRP systems. Hence, the YKJ strain is able to degrade aromatic compounds, which will make it highly suitable for application in bioremediation of polluted environments.

## Materials and Methods

### Bacterium, media and culture conditions

The bacterium under study was previously isolated from an industrial zone in Mahshahr, south of Iran by Jahanshahi *et al*. in 2014. It was identified as a member of the genus *Pseudomonas*, and designated as the *Pseudomonas* YKJ strain, capable of utilizing BPA as a sole carbon and energy source^13^. In order to investigate the degradation of aromatic compounds, *Pseudomonas* sp. YKJ was inoculated into liquid mineral salts medium (1.3 g K_2_HPO_4_.3H_2_O, 1 g (NH_4_)_2_SO_4_, 0.2 g MgSO_4_.7H_2_O, 0.016 g FeCl_3_.6H_2_O, 0.050 g CaCl_2_, 40 g NaCl per litre, and appropriate amounts of the aromatic compounds) and incubated at 30 °C and with shaking at 200 rpm. For preparation of fresh culture, bacteria were streaked onto LB agar medium (10 g tryptone, 5 g yeast extract and 10 g NaCl, 15 g agar per liter) and incubated at 30 °C. Pure cultures obtained on LB agar medium were used for preparation and storage of stock cultures in 30% glycerol at −70 °C.

### DNA extraction

A single colony of the *Pseudomonas* YKJ strain grown on LB agar medium was inoculated into LB broth (10 g tryptone, 10 g NaCl and 5 g yeast extract per litre) and incubated at 30 °C with shaking at 180 rpm for 20 h. Bacterial cells were harvested by centrifugation. Genomic and plasmid DNAs were extracted via the DNA FAST extraction kit (Qiagen, CA, USA) and Fermentas plasmid extraction kit (Fermentas/ThermoFisher Scientific, USA), respectively, and in accordance with manufacturer’s instructions. DNA extraction was confirmed by agarose gel electrophoresis. DNA quality was also determined using a NanoDropTM spectrophotometer (Thermo Scientific NanoDrop 2000c, USA). In order to further evaluate the quality of the genomic DNA, PCR was performed for the 16S rRNA gene using B1 (AGAGTTTGATCCTGGCTTAG) and B2 (TAAGGAGGTGATCCAGC) universal primers^62,63^.

### Workflow for sequencing of the YKJ genome

Bacterial culture, genomic DNA preparation, next-generation sequencing (NGS), *de novo* genome assembly and merging of contigs and subsequent bioinformatics analyses workflow are explained in detail below. Figure 1 presents a flow chart for the complete workflow.

### Genome sequencing, assembly and annotation

The genome sequencing of *Pseudomonas* YKJ was carried out by Macrogen public biotechnology Co., Ltd.(Seoul, South Korea) using the HiSeq 2000 sequencing platform (Illumina Inc., San Diego, CA). The DNA sample was used to produce a pair-end sequencing library with an average size of 350 bp. Low quality sequence data were filtered and trimmed using the FastQC V0.10.1 (https://www.bioinformatics.babraham.ac.uk/projects/fastqc/) and Trimmomatic V0.36 tools (http://www.usadellab.org/cms/index.php?page=trimmomatic). The cleaned reads were then assembled in k-mer lengths of 31, 41, 47, 53, 59, 65, 67, 71, 77, 87 and 91 using the Velvet-1.2.10^64^ and SPAdes 3.9.0 programs^65^. Prodigal-2.6.3 software (http://compbio.ornl.gov/prodigal/) was employed to predict genes. Moreover, the quality of this *de novo* assembly was assessed by BUSCO v3 tools (https://gitlab.com/ezlab/busco). The genome annotation of the ordered assembled genome as well as gene detection were carried out by using the RAST (http://rast.nmpdr.org/) and BlastKOALA v2.1^66^ servers. CDS, tRNA and rRNA features of the genome were also filtered and reported using the FeatureExtract 1.2L Server (http://www.cbs.dtu.dk/services/FeatureExtract/).

### Pathway analysis

The predicted gene sequences were examined to detect the existence of certain metabolic pathways. Hence, the corresponding pathways were completely identified through manual examination of the determined genes functions based on comparisons with the KEGG (Kyoto Encyclopedia of Genes and Genomes) PATHWAY Database^67^. The identified pathways were graphically illustrated using PathVisio 3.2.0, (http://www.pathvisio.org/).

### Phylogenetic analysis

MLST studies^50^ based on analysis of housekeeping genes were performed to identify the genus and species of the YKJ strain. Phylosift v1.0.1 software (http://phylosift.wordpress.com) was also applied to indicate phylogenetic models. Consequently, a phylogenetic tree was reconstructed based on the aligned sequences of whole genomes using the bootstrap method available in CVTree_V3.0 (http://tlife.fudan.edu.cn/cvtree/cvtree3/).

### Whole genome comparisons

Based on phylogenetic analysis results, seven *P*. *pseudoalcaligenes* genomes were selected to identify the closest reference genome using the Bowtie2 v2.1.0 software^68^. Moreover, several genomic features including the number of genes, number of tRNAs and rRNAs, genome size and GC contents of the participating seven strains were compared using the NCBI databases. In order to find the closest reference genome, all reads were mapped to each of the seven *P*.*pseudoalcaligenes* strains, namely CECT5344, NBRC14167, AD6, S1, KF707, KF707_a1 and Pac. The seven genomes of the *P*. *pseudoalcaligenes* strains were aligned and compared with the YKJ draft scaffold assembly using the BRIG 0.95 application (http://brig.sourceforge.net/). We also oriented and compared the genomic sequence of the YKJ strain to that of the *P*. *pseudoalcaligenes* CECT5344 strain using the progressive Mauve program available in the Mauve V2.0 software (http://darlinglab.org/mauve/download.html).

### Pan- and core-genome analysis

Roary v3.0, a prokaryote pan-genome pipeline, (http://sanger-pathogens.github.io/Roary) was used to accomplish Pan- and core- genome analysis of *P*. *pseudoalcaligenes* strains comprising the YKJ and its seven close strains. Additionally, a phylogenetic tree was created using Roary, based on the presence and absence of core and unique genes.

### Plasmid detection and annotation

Using SPAdes v3.9.0 and SSPACE v2.0 (https://github.com/nsoranzo/sspace_basic), plasmid DNA sequence was identified and extracted from the whole sequence. Then, the selected scaffolds were analyzed on the PlasmidFinder (http://cge.cbs.dtu.dk/services/Plamidinder/) and Addgene (https://www.addgene.org/analyze-sequence/) servers. Moreover, the YKJ plasmid sequence was aligned and compared with the closest identified plasmid sequences using the BRIG v0.95 software. In addition, the plasmid DNA was annotated in RAST and Addgene servers.

### Bacterial growth in presence of aromatic compounds

Various concentrations of aromatic compounds were each separately added to 10 ml of mineral salts medium (Fig. S9). The culture media were inoculated with a 3% inoculum obtained from a pre-culture (OD_600_ = 1), and incubated at 30 °C with shaking at 180 rpm. Growth was then assessed spectrophotometrically by measuring the optical density (OD) of the culture samples at 600 nm at specified time intervals (12 hours).

### Bacterial substrate preference test

After determining the suitable concentrations of aromatic compounds for bacterial growth, the aromatic compounds preferred by the YKJ strain, as the best sole source of carbon and energy, were investigated. Therefore, selected concentrations of each aromatic compound was added to 10 ml of mineral salts medium (Fig. S10). The culture media were inoculated and incubated as mentioned above, and bacterial growth was measured at 600 nm at specified time intervals (12 hours), as mentioned previously.

### Antibiotic resistance test

Several experiments were designed to investigate the resistance of the YKJ strain to antibiotics. LB agar medium was prepared with standard concentrations of five different antibiotics, namely ampicillin (100 μg), tetracycline (25 μg), kanamycin (50 μg), rifampicin (100 μg) and chloramphenicol (34 μg). The YKJ strain was then streaked onto LB agar medium containing the antibiotic. Moreover, the extracted pYKJ was transferred into *E*. *coli* DH5α cells using the heat shock method, as described previously^69^. *E*. *coli* transformants were then streaked onto LB agar media and incubated at 37 °C. In the next step, five single colonies of *E*. *coli* DH5α strain grown on LB agar medium were inoculated into five flasks congaing LB broth (10 ml), and incubated at 37 °C, with shaking at 180 rpm for 17 h. Afterwards, plasmid DNA was extracted using the Fermentas plasmid extraction kit (Fermentas/ThermoFisher Scientific, USA). Plasmid DNA extraction was approved by the agarose gel electrophoresis procedure. In addition, 50 µl of each culture medium was streaked onto LB agar plates containing ampicillin and chloramphenicol, so as to investigate antibiotic resistance of the *E*. *coli* transformants containing the pYKJ plasmid.

## Supplementary information

Supporting Information

## Data Availability

All obtained data within this study are included in this paper and its supplementary files. The whole genome sequencing project of *P*. *pseudoalcaligenes* YKJ strain has been announced in https://www.ncbi.nlm.nih.gov under the accession number of PTLV00000000 and BioProject code: PRJNA434488.
