# Supplementary material for: Comprehensive genomic analysis of an indigenous Pseudomonas pseudoalcaligenes degrading phenolic compounds
Source: Sci Rep. 2019 Sep 4;9:12736. doi: 10.1038/s41598-019-49048-6 (PMC6726644; doi:10.1038/s41598-019-49048-6)
Supplement: Supplementary file 1 — Supporting Information [file 41598_2019_49048_MOESM1_ESM.docx]

***Scientific Reports* Supporting Information**

**Comprehensive genomic analysis of an indigenous *Pseudomonas* *pseudoalcaligenes* degrading phenolic compounds**

‬‬‬‬‬‬‬‬‬‬‬‬‬‬‬‬

Maryam Safari ^1,2^, Bagher Yakhchali^1*^, Vahid shariati J^3^

1. Institute of Industrial and Environmental Biotechnology, National Institute of Genetic Engineering and Biotechnology (NIGEB), Tehran, I. R. Iran
2. Department of Biology, Faculty of Science, NourDanesh Institute of Higher Education, Isfahan Province, Meymeh, Danesh Blv, I. R. Iran
3. Plant Molecular Biotechnology Department, National Institute of Genetic Engineering and Biotechnology (NIGEB), Tehran, I. R. Iran

*Corresponding Author:

Bagher Yakhchali, Ph.D.

Institute of Industrial and Environmental Biotechnology, National Institute of Genetic Engineering and Biotechnology, Tehran, Iran.

E-mail: [bahar@nigeb.ac.ir](mailto:bahar@nigeb.ac.ir)

**
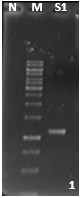

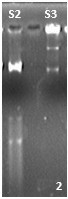
**

**Figure S1.** **1)** Amplification of 16s_rRNA gene. **2)** Genomic and plasmid DNA from *YKJ* isolate. (M: 1 kb molecular weight marker, N: negative control (Distilled water), S1: 16s_rRNA gene, S2: Genomic DNA, S3: plasmid DNA.). Vertically sliced images were cropped from the separate gels namely figures X (S1. 1) and Y (S1. 2) presented as following.


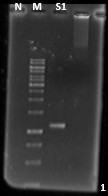


**Figure X (S1.** **1)**


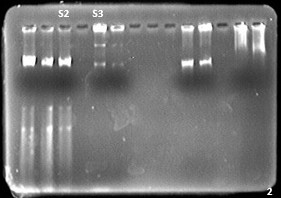


**Figure Y (S1.** **2)**

**
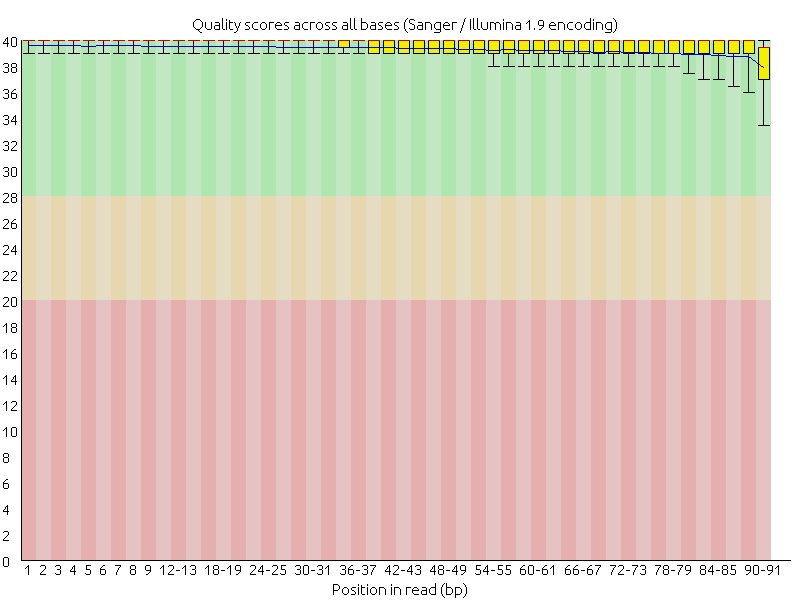
**

**Figure S2** Quality scores across all bases after QC step

**
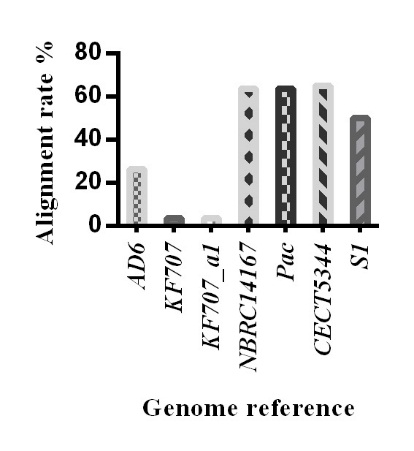
**

**Figure S3** Percentage of reads mapped on the 7 *P.pseudoalcaligenes* strains


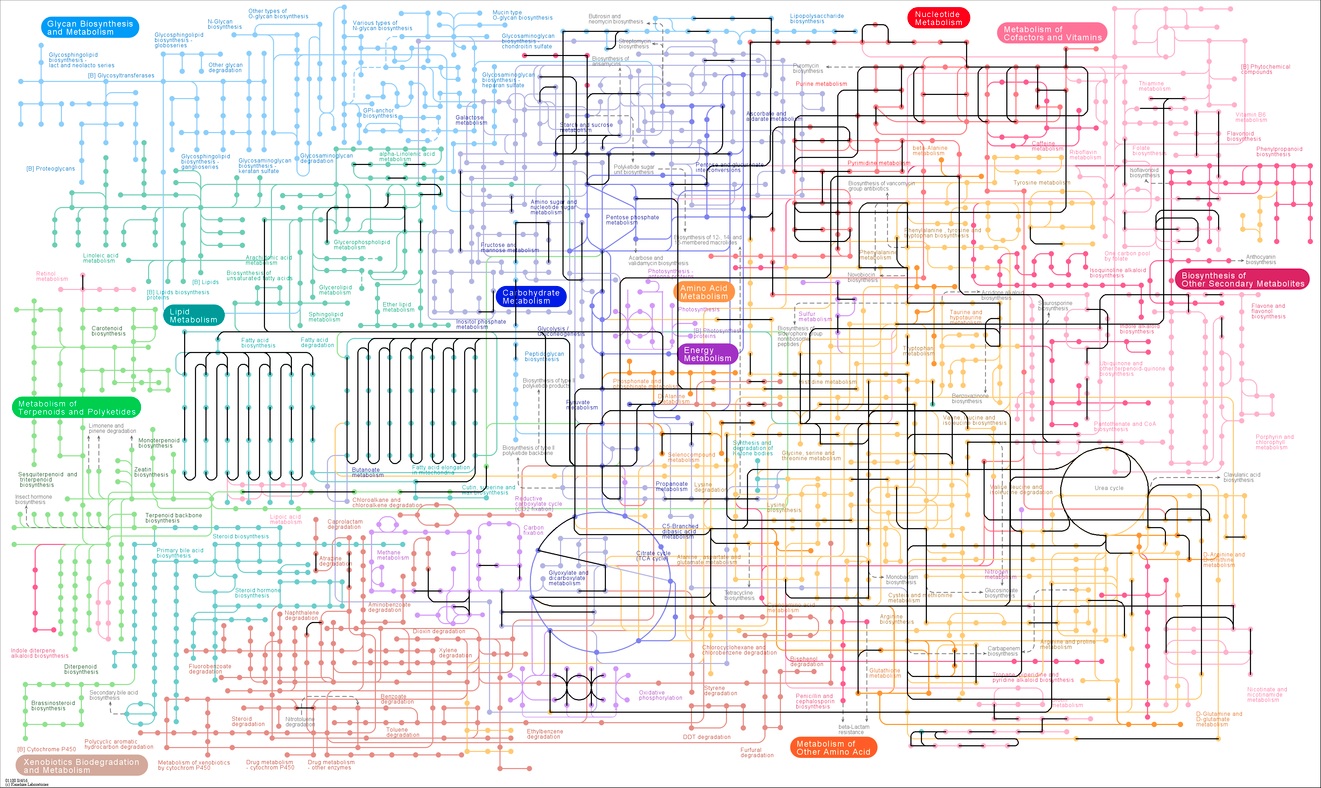


**Figure S4** An overview of biochemical, catabolic and biosynthetic pathways (black circuits) in *YKJ* strain generated by KEGG server (Kanehisa, M., Sato, Y., Furumichi, M., Morishima, K., and Tanabe, M.; New approach for understanding genome variations in KEGG. Nucleic Acids Res. 47, D590-D595 (2019)).


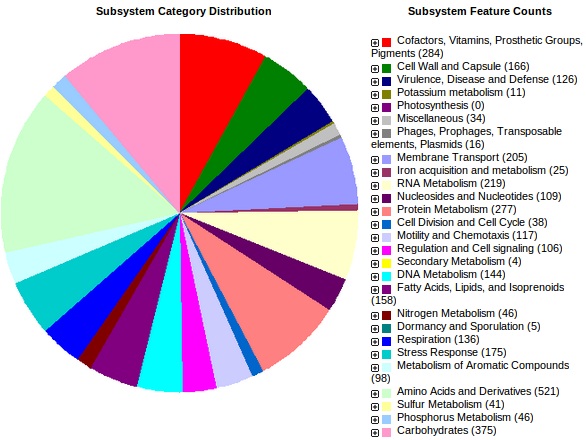


**Figure S5** Metabolic properties of *YKJ* strain generated by RAST server


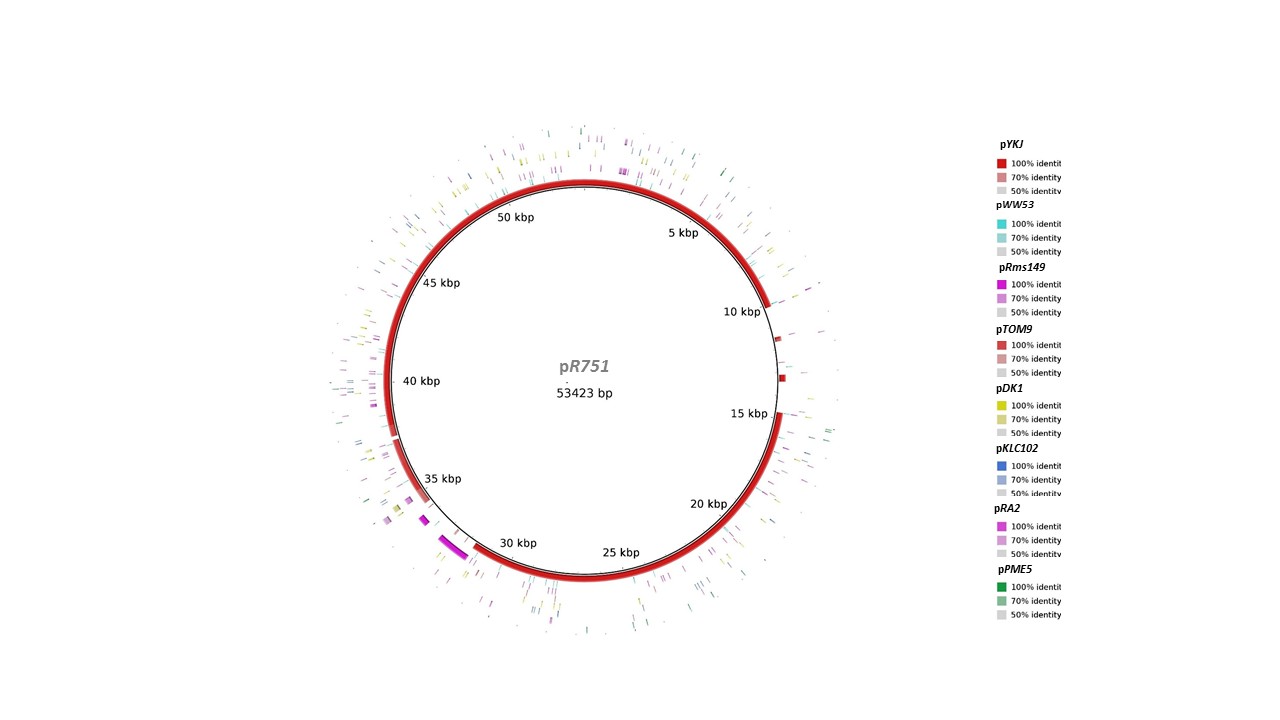


**Figure S6** DNA comparisons of *YKJ* plasmid and *pRms149*, *pTOM9*, *pKLC102*, *pRA2*, *pDK1*, *pWW53 and* *pPME5* against *R751* plasmid (as a reference) portraying by BRIG 0.95, the circular graph shows the whole sequence comparison of *pR751* (inner black circle) with other 8 sequenced plasmids. The intensity of colors reveal the similarity of *pR751* to *pYKJ*.


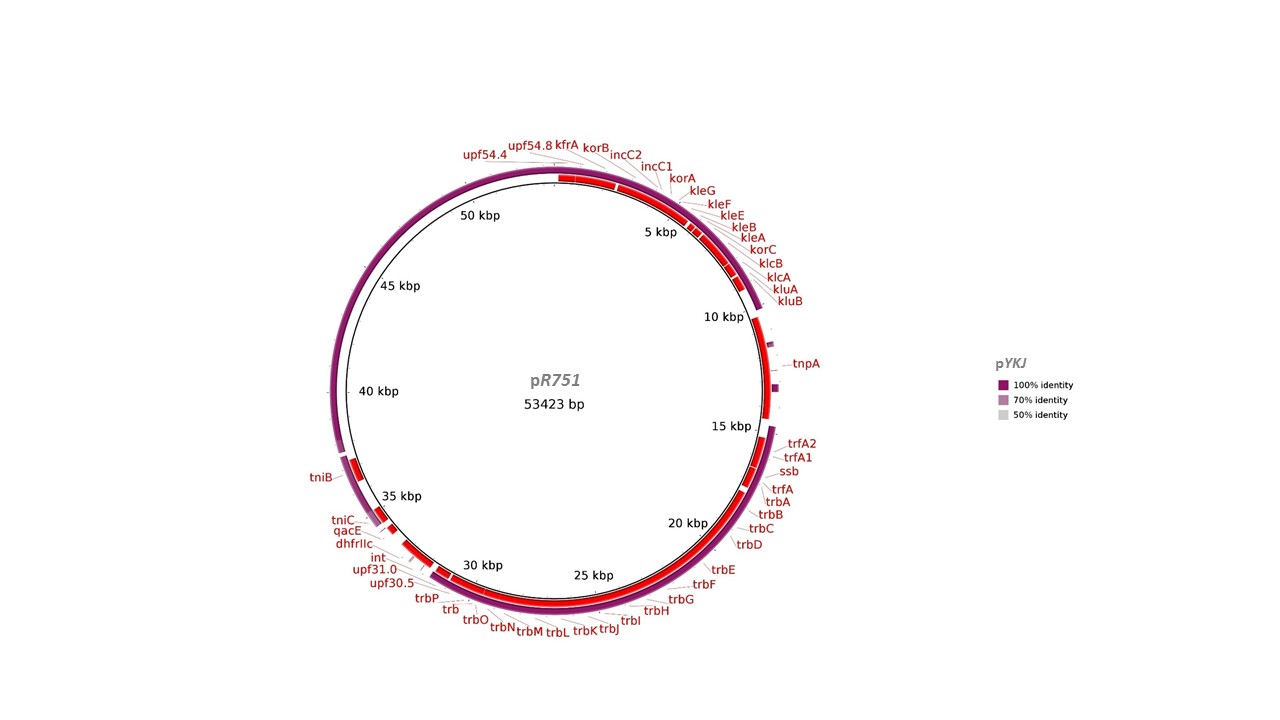


**Figure S7** DNA comparisons of *YKJ* plasmid against *pR751* (as a reference), the inner black and red circles show the whole sequence and GenBank file of *pR751*, sequentially.


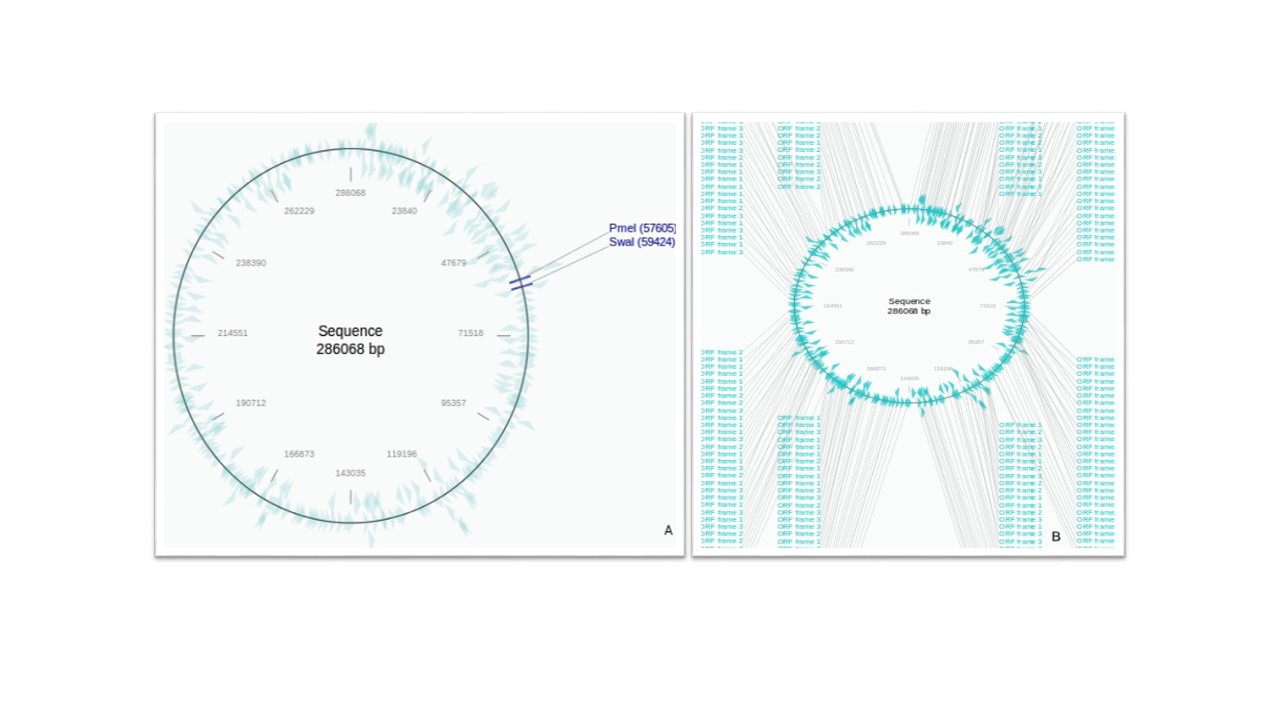


**Figure S8** Display of **A)** cutting position of Pmel and Swal restriction enzymes and **B)** the ORFs template on the plasmid *YKJ* generating by Addgene server.

**
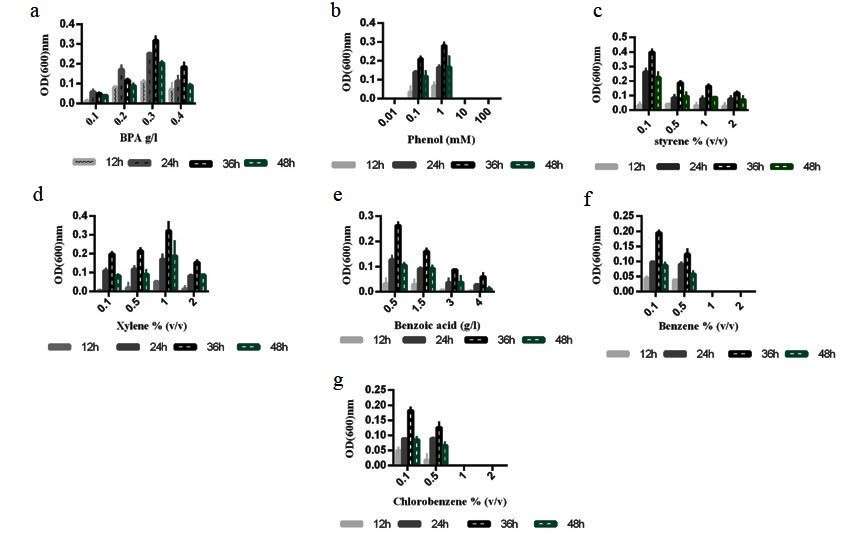
**

**Figure S9** Growth of the *YKJ* strain in presence of pollutant compounds namely a) bisphenol A,b) phenol, c) styrene, d) xylene, e) benzoic acid, f) benzene and g) chlorobenzene

**
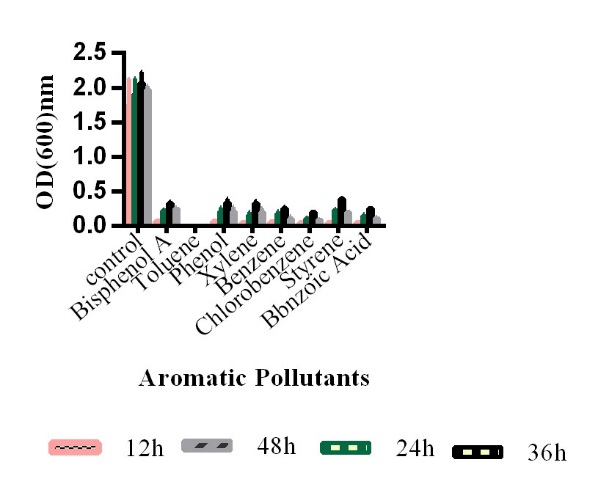
**

**Figure S10** Growth of the *YKJ* strain in presence of various aromatic pollutants namely Bis phenol A (0.3 g/l), Phenol (0.1 mM), Benzoic acid (0.5 g/l), Benzene (0.1 % v/v), Chlorobenzene (0.1 % v/v), Toluene (0.1 % v/v), Styrene (0.1 % v/v) and Xylene (0.1 % v/v). As shown in graph, the *YKJ* strain is more capable of degrading and consuming bisphenol A, phenol and styrene compounds as only source of energy.

**
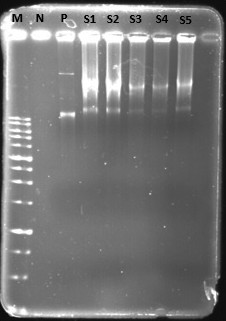
**

**Figure S11** The *YKJ* plasmid. (M: 1 kb molecular weight marker, N: negative control, P: posetive control (p*YKJ* extracted from *p. pseudoalcaligenes* strain *YKJ*), S1, S2, S3, S4 and S5: p*YKJ* extracted from *E.coli DH5α*.)

**Table S1** The results of the quality control step of genomic and plasmid DNA samples

| Sample | Concentration‬ (ng/μl) | λ 260 /λ 280 | λ 260 /λ 230 |
| --- | --- | --- | --- |
| Genomic DNA | 2233.6 | 1.79 | 2.03 |
| plasmid | 151.1 | 1.91 | 2.17 |

**Table S2** Basic Statistics of raw and clean sequences

| Measure | Raw sequence | Clean sequence |
| --- | --- | --- |
| Reads (M) | 6,068,771 | 5,062,080 |
| Bases (b) | 612,945,871 |  |
| Read length (bp) | 101 | 91 |
| Q20(%) | 96.28 | 99.00 |
| GC(%) | 63 | 63 |

**Note:** Read (M): Total Number of Reads in million. Bases(b): Total Number of Bases in base. Q20: Percent of base number calls with quality value of 20 or higher. GC%: The percentage of GC content.

**Table S3** The results of reads assembly by Velvet and SPAdes-3.0.0 softwares in Kmer_ length 65

| Tools | N50 | max_length | Contig.no | Total length (bp) |
| --- | --- | --- | --- | --- |
| Velvet | 27295 | 97841 | 592 | 4647962 |
| SPAdes | 96476 | 307845 | 140 | 4905549 |

**Table S4** Results of a BLAST search of 16s rRNA gene isolated from scaffold *YKJ* in the NCBI GenBank database

| Gene Marker | Database | Species Identified | Score (bits) | E-Value | Identification |
| --- | --- | --- | --- | --- | --- |
| 16_*S rRNA* | 16s ribosomal RNA sequences (Bacteria and Archaea) | *Pseudomonas chengduensis strain MBR* | 1511 | 0.0 | 99% |
|  |  | *Pseudomonas mendocina ymp strain ymp* | 1495 | 0.0 | 99% |
|  |  | *Pseudomonas alcaliphila strain AL15-21* | 1483 | 0.0 | 99% |
|  |  | *Pseudomonas pseudoalcaligenes strain Stanier 63* | 1477 | 0.0 | 99% |
|  |  | seudomonas pseudoalcaligenes strain JCM 5968 | 1473 | 0.0 | 99% |

**Table S5** Phylogenetic affiliation based on MLST analysis

| Gene Markers | Database | Closest speciess | Score (bits) | E-Value | Identification |
| --- | --- | --- | --- | --- | --- |
| *rpoD* | PseudoMLSA | ***Pseudomonas pseudoalcaligenes CECT 5344*** | 1212 | 0.0 | 99% |
|  |  | *Pseudomonas alcaliphila 34 IC* | 1024 | 0.0 | 96% |
|  |  | *Pseudomonas mendocina DLHK* | 986 | 0.0 | 95% |
| *rpoB* | PseudoMLSA | ***Pseudomonas pseudoalcaligenes CECT 5344*** | 3948 | 0.0 | 98% |
|  |  | *Pseudomonas alcaliphila 34 IB* | 3684 | 0.0 | 96% |
|  |  | *Pseudomonas mendocina S5.2* | 3556 | 0.0 | 96% |
| *gyrB* | PseudoMLSA | ***Pseudomonas pseudoalcaligenes CECT 5344*** | 2289 | 0.0 | 98% |
|  |  | *Pseudomonas alcaliphila 34 ID* | 1837 | 0.0 | 94% |
|  |  | *Pseudomonas mendocina DLHK* | 1361 | 0.0 | 89% |
| 16*s_rRNA* | PseudoMLSA | ***Pseudomonas pseudoalcaligenes CECT 5344*** | 1526 | 0.0 | 99% |
|  |  | *Pseudomonas alcaliphila 34* | 1526 | 0.0 | 99% |
|  |  | *Pseudomonas mendocina DLHK* | 1520 | 0.0 | 99% |

**Table S6** Percentage of reads mapped on the closest reference genome (*P.pseudoalcaligenes* strain *CECT5344)*

| Sample | No. of reads | Total no. of  bases | Percentage of reads  passed filtering | Longest read  (bases) | longest |
| --- | --- | --- | --- | --- | --- |
| *YKJ*_clean_filtered_1.fq | 5,062,080 | 511,270,080 | 100.00% | 91 bases | 91 bases |
| *YKJ*_clean_filtered_2.fq | 5,062,080 | 511,270,080 | 100.00% | 91 bases | 91 bases |
| Total | 10,124,160 | 1,022,540,160 | 100.00% | 91 bases | 101 bases |

**Table S7** Comparative genome analysis

| Strains | Genome size (Mb) | GC % | Scaffolds | No. of rRNA | No. of tRNA | No. of Gene | | No. of Protein | GenBank  Accession |
| --- | --- | --- | --- | --- | --- | --- | --- | --- | --- |
| *YKJ* | 4.9 | 63 | 140 | 3 | 48 | 4583 | 4565 | | [GCA_002941105.1](https://www.ncbi.nlm.nih.gov/assembly/GCA_002941105.1) |
| *CECT5344* | 4.69 | 62.3 | 1 | 12 | 68 | 4514 | 4115 | | GCA_00029707./2 |
| *Pac* | 4.69 | 62.3 | 1 | 12 | 68 | 4521 | 4134 | | GCA_000953455.1 |
| *KF707* | 6.18 | 65.5 | 2 | 15 | 73 | 5722 | 5533 | | GCA-002355475.1 |
| *S1* | 6.89 | 62.4 | 131 | 8 | 78 | 6636 | 6042 | | GCA-000962895.1 |
| *AD6* | 5.39 | 62.5 | 198 | … | 70 | 4934 | 4821 | | GCA_000626735.1 |
| *NBRC14167* | 4.7 | 62.3 | 204 | 6 | 57 | 4620 | 4168 | | GCA_002091775.1 |
| *KF707_a1* | 6.68 | 65.3 | 229 | 27 | 73 | 6211 | 6111 | | GCA_000262065.3 |

**Table S8** The results of the pan- and Core-genome analysis for *YKJ* strain

| *YKJ* | *S1* | *Pac* | *NBRC14167* | *KF707_a1* | *KF707* | *CECT5344* | *AD6* | Ko no. | Annotation | Genes | Trait |
| --- | --- | --- | --- | --- | --- | --- | --- | --- | --- | --- | --- |
| 1 | 0 | 0 | 0 | 0 | 0 | 0 | 0 | K00484 | Nitrilotriacetate monooxygenase  component B | *hpaC* | Oxidoreductases |
| 1 | 0 | 0 | 0 | 0 | 0 | 0 | 0 | K00249 | Acyl-CoA dehydrogenase | *ACADM* |  |
| 1 | 0 | 0 | 0 | 0 | 0 | 0 | 0 | K07729 | putative transcriptional regulator | *...* | Transcription factors |
| 1 | 0 | 0 | 0 | 0 | 0 | 0 | 0 | K21756 | HTH-type transcriptional regulator CatM | *catM* |  |
| 1 | 0 | 0 | 0 | 0 | 0 | 0 | 0 | K21699 | HTH-type transcriptional regulator TfdS | *tfdS* |  |
| 1 | 0 | 0 | 0 | 0 | 0 | 0 | 0 | K05817 | DNA-binding transcriptional regulator HcaR | *hcaR* |  |
| 1 | 0 | 0 | 0 | 0 | 0 | 0 | 0 | ... | Transcriptional repressor RcnR | *rcnR* |  |
| 1 | 0 | 0 | 0 | 0 | 0 | 0 | 0 | K02444 | Glycerol-3-phosphate regulon repressor | *glpR_1* |  |
| 1 | 0 | 0 | 0 | 0 | 0 | 0 | 0 | K21826 | HTH-type transcriptional regulator CdhR | *...* |  |
| 1 | 0 | 0 | 0 | 0 | 0 | 0 | 0 | K13040 | sensor histidine kinase TtrS | *ttrS* | Two-component system |
| 1 | 0 | 0 | 0 | 1 | 1 | 0 | 1 | K13041 | two-component response regulator, LuxR family | *ttrR* |  |
| 1 | 0 | 0 | 0 | 0 | 0 | 0 | 0 | K08358 | Tetrathionate reductase subunit B precursor | *ttrB* |  |
| 1 | 0 | 0 | 0 | 0 | 0 | 0 | 0 | K08359 | Tetrathionate reductase subunit C | *ttrC* |  |
| 1 | 0 | 0 | 0 | 0 | 0 | 0 | 0 | K08357 | Tetrathionate reductase subunit A precursor | *ttrA* |  |
| 1 | 0 | 0 | 0 | 0 | 0 | 0 | 0 | K06137 | pyrroloquinoline-quinone synthase | *PqqC_2* | ... |
| 1 | 0 | 0 | 0 | 0 | 0 | 0 | 0 | K06136 | Coenzyme PQQ synthesis protein B | *pqqB_2* |  |
| 1 | 0 | 0 | 0 | 0 | 0 | 0 | 0 | K18989 | Toluene efflux pump membrane transporter TtgE | *ttgE* | Transporters |
| 1 | 0 | 0 | 0 | 0 | 0 | 0 | 0 | K02440 | Glycerol uptake facilitator protein | *glpF* |  |
| 1 | 0 | 0 | 0 | 0 | 0 | 0 | 0 | K03585 | Solvent efflux pump periplasmic linker SrpA precursor | *srpA_2* |  |
| 1 | 0 | 0 | 0 | 0 | 0 | 0 | 0 | K11042 | superantigen-like protein | *set* | ... |
| 1 | 0 | 0 | 0 | 0 | 0 | 0 | 0 | K01761 | O-succinylhomoserine sulfhydrylase | *metZ_1* | Lyases |
| 1 | 0 | 0 | 0 | 0 | 0 | 0 | 0 | K14060 | Putative DNA-invertase from lambdoid prophage Rac | *pinR* | ... |
| 1 | 0 | 0 | 0 | 0 | 0 | 0 | 0 | K03657 | ATP-dependent DNA helicase UvrD1 | *uvrD1* | Hydrolases |
| 1 | 0 | 0 | 0 | 0 | 0 | 0 | 0 | K00784 | putative metal-dependent hydrolase | *rnz* |  |
| 1 | 0 | 0 | 0 | 0 | 0 | 0 | 0 | K07319 | N-6 DNA Methylase | *yhdJ* | Methyltransferases |
| 1 | 0 | 0 | 0 | 0 | 0 | 0 | 0 | K03523 | Biotin transporter BioY2 | *bioY2* | ABC transporters |
| 1 | 0 | 0 | 0 | 0 | 0 | 0 | 0 | K07473 | Antitoxin DinJ | *dinJ* | Toxin-antitoxin system |
| 1 | 0 | 0 | 0 | 0 | 0 | 0 | 0 | ... | Antitoxin VapB11 | *...* |  |
| 1 | 0 | 0 | 0 | 0 | 0 | 0 | 0 | ... | Ribonuclease VapC11 | *...* |  |
| 1 | 0 | 0 | 0 | 0 | 0 | 0 | 0 | K00864 | Glycerol kinase | *glpK_1* | Phosphotransferases |
| 1 | 0 | 0 | 0 | 0 | 0 | 0 | 0 | K00868 | Pyridoxine kinase | *pdxK* |  |
| 1 | 0 | 0 | 0 | 0 | 0 | 0 | 0 | K20791 | N-alpha-acetyltransferase | *ttr* | Acetyl transferase |
| 1 | 0 | 0 | 0 | 0 | 0 | 0 | 0 | K01626 | Phospho-2-dehydro-3-deoxyheptonate aldolase, Tyr-sensitive | *aroF* | Alkyl transferase |
| 1 | 0 | 0 | 0 | 0 | 0 | 0 | 0 | K11433 | SET domain and mariner transposase fusion gene | *...* | Putative transposase |
| 1 | 0 | 0 | 0 | 0 | 0 | 0 | 0 | K07481 | IS5 transposase and trans-activator | *..* |  |
| 1 | 0 | 0 | 0 | 0 | 0 | 0 | 0 | K07497 | IS2 transposase TnpB | *insD1* |  |
| 1 | 0 | 0 | 0 | 0 | 0 | 0 | 0 | K07484 | Transposase IS66 family protein | *...* |  |
| 1 | 0 | 0 | 0 | 0 | 0 | 0 | 0 | K13607 | E-cinnamoyl-CoA:R-phenyllactate CoA transferase | *fldA* | CoA-transferases |
| 1 | 0 | 0 | 0 | 0 | 0 | 0 | 0 | K07340 | Inner membrane protein YbbJ | *...* | ... |
| 1 | 0 | 0 | 0 | 0 | 0 | 0 | 0 | K02317 | Primosomal protein DnaI | *dnaI* | DNA replication protein |
| 1 | 1 | 0 | 0 | 1 | 1 | 0 | 1 | K03381 | catechol 1,2-dioxygenase | *catA* | Ortho-cleavage pathway |
| 1 | 1 | 0 | 0 | 1 | 1 | 0 | 1 | K01856 | muconate cycloisomerase | *catB* |  |
| 1 | 1 | 0 | 0 | 1 | 1 | 0 | 1 | K03464 | muconolactone D-isomerase | ***catC*** |  |
| 1 | 1 | 0 | 0 | 1 | 1 | 0 | 1 | K01055 | 3-oxoadipate enol-lactonase | *pcaD* |  |
| 1 | 1 | 0 | 0 | 1 | 1 | 0 | 1 | K14727 | 4-carboxymuconolactone decarboxylase | *pcaL* |  |
| 1 | 1 | 0 | 0 | 0 | 1 | 0 | 0 | K00446 | catechol 2,3-dioxygenase | *dmpB(xylE)* | Meta-cleavage pathway |
| 1 | 0 | 0 | 0 | 0 | 0 | 0 | 0 | K07104 | catechol 2,3-dioxygenase | *catE* |  |
| 1 | 1 | 1 | 0 | 0 | 1 | 1 | 0 | K10217 | Aminomuconate-semialdehyde | *DmpC (xylG)* |  |
| 1 | 0 | 0 | 0 | 0 | 0 | 0 | 0 | K01821 | 4-oxalocrotonate tautomerase | *praC* |  |
| 1 | 0 | 0 | 0 | 0 | 0 | 0 | 0 | K01617 | 2-oxo-3-hexenedioate decarboxylase | *xylI* |  |
| 1 | 1 | 0 | 0 | 1 | 1 | 0 | 0 | K10216 | 2-hydroxymuconate-semialdehyde hydrolase | *DmpD*  *(xylF)* |  |
| 1 | 1 | 1 | 0 | 1 | 1 | 1 | 0 | K18364 | 2-oxopent-4-enoate | *bphH(xylJ)* |  |
| 1 | 0 | 0 | 0 | 0 | 0 | 0 | 1 | K02554 | 2-keto-4-pentenoate hydratase | *dmpH (mhpD)* |  |
| 1 | 0 | 0 | 0 | 1 | 1 | 0 | 0 | K18365 | 4-hydroxy-2-oxohexanoate aldolase | *bphI* |  |
| 1 | 1 | 1 | 0 | 1 | 1 | 1 | 1 | K01666 | 4-hydroxy 2-oxovalerate aldolase | *mhpE*  *(dmpG)* |  |
| 1 | 0 | 0 | 0 | 1 | 1 | 0 | 0 | K18366 | propanal dehydrogenase | *bphJ* |  |
| 1 | 1 | 1 | 0 | 1 | 1 | 1 | 1 | K04073 | acetaldehyde dehydrogenase | *mhpF* |  |
| 1 | 1 | 1 | 1 | 0 | 0 | 1 | 1 | K15342 | CRISPR-associated protein Cas1 | *cas1 (ygbT)* | CRISPR-Cas system |
| 1 | 0 | 1 | 1 | 0 | 0 | 1 | 0 | K09951 | CRISPR-associated protein Cas2 | *cas2 (ygbF)* |  |
| 1 | 1 | 1 | 1 | 0 | 0 | 1 | 1 | K07012 | CRISPR-associated helicase Cas3 | *cas3 (ygcB)* |  |
| 1 | 0 | 0 | 0 | 0 | 0 | 0 | 0 | K07464 | CRISPR-associated exonuclease Cas4 | *cas4* |  |
| 1 | 0 | 1 | 1 | 0 | 0 | 1 | 0 | K19123 | CRISPR system Cascade subunit CasA | *casA (cse1)* |  |
| 1 | 0 | 1 | 1 | 0 | 0 | 1 | 0 | K19046 | CRISPR-associated protein, Cse2 family | *casB (cse2)* |  |
| 1 | 0 | 1 | 1 | 0 | 0 | 1 | 0 | K19126 | CRISPR system Cascade subunit CasE | *casE (cse3)* |  |
| 1 | 0 | 1 | 1 | 0 | 0 | 1 | 0 | K19124 | CRISPR system Cascade subunit CasC | *casC (cse4)* |  |
| 1 | 0 | 1 | 1 | 0 | 0 | 1 | 0 | K19125 | CRISPR system Cascade subunit CasD | *casD (cse5)* |  |
| 1 | 0 | 0 | 0 | 0 | 0 | 0 | 0 | K19117 | CRISPR-associated protein, Csd1 family | *csd1 (cas8c)* |  |
| 1 | 0 | 0 | 0 | 0 | 0 | 0 | 0 | K19118 | CRISPR-associated protein Csd2 | *csd2 (cas7)* |  |
| 1 | 0 | 0 | 0 | 0 | 0 | 0 | 0 | K19119 | CRISPR-associated protein Cas5d | *cas5d* |  |

**Table S9** Gene annotation of *P. pseudoalcaligenes* *YKJ* genome based on genes contributed in ortho- and meta-cleavage pathways

| Traits | KEGG  Gene Name | Ko  number | Activity | EC  number |
| --- | --- | --- | --- | --- |
| Catechol ortho-cleavage genes | *catA* | K03381 | catechol 1,2-dioxygenase | 1.13.11.1 |
|  | *catB* | K01856 | muconate cycloisomerase | 5.5.1.1 |
|  | *catC* | K03464 | muconolactone D-isomerase | 5.3.3.4 |
|  | *pcaD* | K01055 | 3-oxoadipate enol-lactonase | 3.1.1.24 |
|  | *pcaL* | K14727 | 4-carboxymuconolactone decarboxylase | 4.1.1.44 |
| Catechol meta-cleavage genes | *dmpB* (*xylE*) | K00446 | catechol 2,3-dioxygenase | 1.13.11.2 |
|  | *catE* | K07104 | catechol 2,3-dioxygenase | 1.13.11.2 |
|  | *dmpC* | K10217 | Aminomuconate-semialdehyde | 1.2.1.32 |
|  | *praB* (*xylG*) |  | 2-hydroxymuconate-6-semialdehyde dehydrogenase | 1.2.1.85 |
|  | *dmpI* (*xylH*) | K01821 | 4-oxalocrotonate tautomerase / 4-oxalocrotonate isomerase | 5.3.2.6 |
|  | *dmpH* (*xylI*) | K01617 | 2-oxo-3-hexenedioate decarboxylase | 4.1.1.77 |
|  | *dmpD* (*xylF*) | K10216 | 2-hydroxymuconate-semialdehyde hydrolase | 3.7.1.9 |
|  | *dmpE* (*xylJ*) | K18364 | 2-oxopent-4-enoate hydratase | 4.2.1.80 |
|  | *tesE* |  | cis-2-oxohex-4-enoate hydratase | 4.2.1.132 |
|  | *dmpH* (*mhpD*) | K02554 | 2-keto-4-pentenoate hydratase | 4.2.1.80 |
|  | *bphI* (*xylK*) | K18365 | 4-hydroxy-2-oxohexanoate aldolase | 4.1.3.43 |
|  | *dmpG* (*mhpE*) | K01666 | 4-hydroxy 2-oxovalerate aldolase | 4.1.3.39 |
|  | *BphJ* | K18366 | propanal dehydrogenase | 1.2.1.87 |
|  | *mhpF* | K04073 | acetaldehyde dehydrogenase | 1.2.1.10 |

**Table S10** Gene annotation of *P. pseudoalcaligenes* strain *YKJ* genome based on genes involved in aromatic compounds degradation pathways

| Definition | KEGG  Gene Name | EC  number | Ko number | KEGG  Pathway | |
| --- | --- | --- | --- | --- | --- |
| S-(hydroxymethyl)glutathione dehydrogenase / alcohol dehydrogenase | *ADH5* | EC 1.1.1.284 / 1.1.1.1 | K00121 | [Degradation of aromatic compounds](http://www.genome.jp/kegg-bin/show_pathway?1472805441145836/ko01220.args) | |
| catechol 2,3-dioxygenase | *dmpB (xylE)* | EC 1.13.11.2 | K00446 |  |  |
| protocatechuate 3,4-dioxygenase, alpha subunit | *pcaG* | EC 1.13.11.3 | K00448 |  |  |
| protocatechuate 3,4-dioxygenase, beta subunit | *pcaH* | EC 1.13.11.3 | K00449 |  |  |
| p-hydroxybenzoate 3-monooxygenase | *pobA* | EC 1.14.13.2 | K00481 |  |  |
| 3-oxoadipate enol-lactonase | *pcaD* | EC 3.1.1.24 | K01055 |  |  |
| 4-carboxymuconolactone decarboxylase | *pcaC* | EC 4.1.1.44 | K01607 |  |  |
| 2-oxo-3-hexenedioate decarboxylase | *xylI* | EC 4.1.1.77 | K01617 |  |  |
| 4-hydroxy 2-oxovalerate aldolase | *dmpG*  *(mhpE)* | EC 4.1.3.39 | K01666 |  |  |
| 4-oxalocrotonate tautomerase / 4-oxalocrotonate isomerase | *dmpI (xylH)* | EC 5.3.2.6 | K01821 |  |  |
| muconate cycloisomerase | *catB* | EC 5.5.1.1 | K01856 |  |  |
| 3-carboxy-cis,cis-muconate cycloisomerase | *pcaB* | EC 5.5.1.2 | K01857 |  |  |
| catechol 1,2-dioxygenase | *catA* | EC 1.13.11.1 | K03381 |  |  |
| muconolactone D-isomerase | *catC* | EC 5.3.3.4 | K03464 |  |  |
| acetaldehyde dehydrogenase | *mhpF* | EC 1.2.1.10 | K04073 |  |  |
| benzoate/toluate 1,2-dioxygenase subunit alpha | *benA-xylX* | EC 1.14.12.10 / 1.14.12.- | K05549 |  |  |
| benzoate/toluate 1,2-dioxygenase subunit beta | *benB-xylY* | EC 1.14.12.10 / 1.14.12.- | K05550 |  |  |
| dihydroxycyclohexadiene carboxylate dehydrogenase | *benD-xylL* | EC 1.3.1.25 | K05783 |  |  |
| benzoate/toluate 1,2-dioxygenase reductase component | *benC-xylZ* | EC 1.18.1.- | K05784 |  |  |
| 2-hydroxymuconate-semialdehyde hydrolase | *dmpD* | EC 3.7.1.9 | K10216 |  |  |
| 2-hydroxymuconate-6-semialdehyde dehydrogenase | *xylG (praB)* | EC 1.2.1.85 | K10217 |  |  |
| 2-hydroxymuconic semialdehyde dehydrogenase | *dmpC* | EC 1.2.1.32 |  |  |  |
| alcohol dehydrogenase, propanol-preferring | *adhP* | EC 1.1.1.1. | K13953 |  |  |
| NADP-dependent aldehyde dehydrogenase | *aldH* | EC 1.2.1.4 | K14519 |  |  |
| phenol hydroxylase P3 protein | *dmpN* | EC 1.14.13.- | K16242 |  |  |
| Phenol hydroxylase P1 protein | *dmpL* | ... | K16243 |  |  |
| phenol hydroxylase P2 protein | *dmpM* | ... | K16244 |  |  |
| phenol hydroxylase P4 protein | *dmpO* | ... | K16245 |  |  |
| phenol hydroxylase P5 protein | *dmpP* | ... | K16246 |  |  |
| phenol hydroxylase P0 protein | *dmpK* | ... | K16249 |  |  |
| 2-oxopent-4-enoate hydratase | *dmpE (xylJ)* | EC 4.2.1.80 | K18364 |  |  |
| cis-2-oxohex-4-enoate hydratase | *tesE* | EC 4.2.1.132 |  |  |  |
| glutaryl-CoA dehydrogenase | *gcdH* | EC 1.3.8.6 | K00252 | [Benzoate degradation](http://www.genome.jp/kegg-bin/show_pathway?1472805441145836/ko00362.args) | Xenobiotics biodegradation and metabolism |
| catechol 2,3-dioxygenase | *xylE* | EC 1.13.11.2 | K00446 |  |  |
| protocatechuate 3,4-dioxygenase, alpha subunit | *pcaG* | EC 1.13.11.3 | K00448 |  |  |
| protocatechuate 3,4-dioxygenase, beta subunit | *pcaH* | EC 1.13.11.3 | K00449 |  |  |
| p-hydroxybenzoate 3-monooxygenase | *pobA* | EC 1.14.13.2 | K00481 |  |  |
| acetyl-CoA C-acetyltransferase | *atoB* | EC 2.3.1.9 | K00626 |  |  |
| acetyl-CoA acyltransferase | *fadA* | EC 2.3.1.16 | K00632 |  |  |
| 3-oxoadipate enol-lactonase | *pcaD* | EC 3.1.1.24 | K01055 |  |  |
| 4-carboxymuconolactone decarboxylase | *pcaC* | EC 4.1.1.44 | K01607 |  |  |
| 2-oxo-3-hexenedioate decarboxylase | *xylI* | EC 4.1.1.77 | K01617 |  |  |
| 4-hydroxy 2-oxovalerate aldolase | *mhpE*  *(dmpG)* | EC 4.1.3.39 | K01666 |  |  |
| enoyl-CoA hydratase | *echA* | EC 4.2.1.17 | K01692 |  |  |
| 3-hydroxyacyl-CoA dehydrogenase | *fadJ* | EC 1.1.1.35 | K01782 |  |  |
| 4-oxalocrotonate tautomerase / 4-oxalocrotonate isomerase | *dmpI (xylH)* | EC 5.3.2.6 | K01821 |  |  |
| 3-hydroxyacyl-CoA dehydrogenase | *fadB* | EC 1.1.1.35 | K01825 |  |  |
| 3-hydroxybutyryl-CoA epimerase | *fadB* | EC 5.1.2.3 |  |  |  |
| muconate cycloisomerase | *catB* | EC 5.5.1.1 | K01856 |  |  |
| 3-carboxy-cis,cis-muconate cycloisomerase | *pcaB* | EC 5.5.1.2 | K01857 |  |  |
| catechol 1,2-dioxygenase | *catA* | EC 1.13.11.1 | K03381 |  |  |
| muconolactone D-isomerase | *catC* | EC 5.3.3.4 | K03464 |  |  |
| acetaldehyde dehydrogenase | *mhpF* | EC 1.2.1.10 | K04073 |  |  |
| benzoate 1,2-dioxygenase subunit alpha | *benA-xylX* | EC 1.14.12.10 / 1.14.12.- | K05549 |  |  |
| Benzoate 1,2-dioxygenase subunit beta | *benB-xylY* | EC 1.14.12.10 / 1.14.12.- | K05550 |  |  |
| dihydroxycyclohexadiene carboxylate dehydrogenase (benzoate cis-diol dehydrogenase) | *benD-xylL* | EC 1.3.1.25 | K05783 |  |  |
| benzoate 1,2-dioxygenase reductase component | *benC-xylZ* | EC 1.18.1.- | K05784 |  |  |
| 2-hydroxymuconate-semialdehyde hydrolase | *dmpD(xylF)* | EC 3.7.1.9 | K10216 |  |  |
| 2-hydroxymuconate-6-semialdehyde dehydrogenase | *xylG (praB)* | EC 1.2.1.85 | K10217 |  |  |
| 2-hydroxymuconic semialdehyde dehydrogenase | *dmpC* | EC 1.2.1.32 |  |  |  |
| phenol hydroxylase P3 protein | *dmpN* | EC 1.14.13.- | K16242 |  |  |
| phenol hydroxylase P0 protein | *dmpK* | ... | K16249 |  |  |
| 2-oxopent-4-enoate hydratase | *dmpE (xylJ)* | EC 4.2.1.80 | K18364 |  |  |
| cis-2-oxohex-4-enoate hydratase | *tesE* | EC 4.2.1.132 |  |  |  |
| ssuccinyl CoA synthetase beta subunit | *csc* | EC 6.2.1.4 | K01900 |  |  |
| Succinate CoA ligase | *LSC2* | EC 6.2.1.5 |  |  |  |
| Succinate dehydrogenase | *SDHB* | EC 1.3.5.1 | K00235 |  |  |
| 2-ketoglutarate dehydrogenase | *OGDH* | EC 1.2.4.2 | K00164 |  |  |
| isocitrate dehydrogenase | *IDH1* | EC 1.1.1.42 | K00031 |  |  |
| Aconitate hydratase | *acnA* | EC 4.2.1.3 | K01681 |  |  |
| Beta-ketoadipyl CoA thiolase | *paaJ* | EC 2.3.1.174 | K02615 |  |  |
| β-ketoadipate succinyl-CoA transferase | *OXCT* | EC 2.8.3.5 | K01027 |  |  |
| catechol 1,2-dioxygenase | *catA* | EC 1.13.11.1 | K03381 | chlorobenzene degradation |  |
| catechol 2,3-dioxygenase | *dmpB* | EC 1.13.11.2 | K00446 |  |  |
| muconate cycloisomerase | *catB* | EC 5.5.1.1 | K01856 |  |  |
| chloromuconate cycloisomerase | *...* | EC 5.5.1.7 | K01860 |  |  |
| phenol hydroxylase P0 protein | *dmpK* | ... | K16249 |  |  |
| dienelactone hydrolase | *...* | EC 3.1.1.45 | K01061 |  |  |
| phenylacetaldehyde dehydrogenase | *feaB* | EC 1.2.1.39 | K00146 | Styrene degradation |  |
| catechol 1,2-dioxygenase | *catA* | EC 1.13.11.1 | K03381 |  |  |
| catechol 2,3-dioxygenase | *xylE* | EC 1.13.11.2 | K00446 |  |  |
| homogentisate 1,2-dioxygenase | *hmgA* | EC 1.13.11.5 | K00451 |  |  |
| propionate CoA-transferase | *pct* | EC 2.8.3.1 | K01026 |  |  |
| glutaconate CoA-transferase, subunit A | *gctA* | EC 2.8.3.12 | K01039 |  |  |
| glutaconate CoA-transferase, subunit B | *gctB* | EC 2.8.3.12 | K01040 |  |  |
| amidase | *amiE* | EC 3.5.1.4 | K01426 |  |  |
| 2-hydroxymuconate-semialdehyde hydrolase | *dmpD(xylF)* | EC 3.7.1.9 | K10216 |  |  |
| fumarylacetoacetate (FAA) hydrolase | *faaH* | EC 3.7.1.2 | K16171 |  |  |
| muconate cycloisomerase | *catB* | EC 5.5.1.1 | K01856 | Toluene degradation |  |
| catechol 1,2-dioxygenase | *catA* | EC 1.13.11.1 | K03381 |  |  |
| phenol hydroxylase P3 protein | *dmpN* | EC 1.14.13.- | K16242 |  |  |
| phenol hydroxylase P0 protein | *dmpK* | ... | K16249 |  |  |
| catechol 2,3-dioxygenase | *xylE* | EC 1.13.11.2 | K00446 | Xylene degradation |  |
| 2-oxo-3-hexenedioate decarboxylase | *xylI* | EC 4.1.1.77 | K01617 |  |  |
| 4-hydroxy 2-oxovalerate aldolase | *dmpG (mhpE)* | EC 4.1.3.39 | K01666 |  |  |
| 4-oxalocrotonate tautomerase / 4-oxalocrotonate isomerase | *xylH (dmpI)* | EC 5.3.2.6 | K01821 |  |  |
| acetaldehyde dehydrogenase | *dmpF (mhpF)* | EC 1.2.1.10 | K04073 |  |  |
| benzoate/toluate 1,2-dioxygenase subunit alpha | *xylX (benA)* | EC 1.14.12.10 / 1.14.12.- | K05549 |  |  |
| benzoate/toluate 1,2-dioxygenase subunit beta | *xylY (benB)* | EC 1.14.12.10 / 1.14.12.- | K05550 |  |  |
| dihydroxycyclohexadiene carboxylate dehydrogenase | *xylL (benD)* | EC 1.3.1.25 / 1.3.1.67 | K05783 |  |  |
| benzoate/toluate 1,2-dioxygenase reductase component | *xylZ (benC)* | EC 1.18.1.- | K05784 |  |  |
| 2-hydroxymuconate semialdehyde hydrolase | *xylF (dmpD)* | EC 3.7.1.9 | K10216 |  |  |
| 2-hydroxymuconate-6-semialdehyde dehydrogenase | *xylG (praB)* | EC 1.2.1.85 | K10217 |  |  |
| 2-hydroxymuconic semialdehyde dehydrogenase | *dmpC* | EC 1.2.1.32 |  |  |  |
| 2-oxopent-4-enoate hydratase | *xylJ (dmpE)* | EC 4.2.1.80 | K18364 |  |  |
| cis-2-oxohex-4-enoate hydratase | *tesE* | EC 4.2.1.132 |  |  |  |
| 4-hydroxy-2-oxovalerate/4-hydroxy-2-oxohexanoate aldolase | *xylK (bphI)* | EC 4.1.3.43 | K18365 |  |  |
| acetaldehyde dehydrogenase | *xylQ* | EC 1.2.1.10 | K18366 |  |  |
| phenol hydroxylase P0 protein | *dmpK* | ... | K16249 | Phenol degradation |  |
| catechol 2,3-dioxygenase | *dmpB (xylE)* | EC 1.13.11.2 | K00446 |  |  |
| 2-hydroxymuconate semialdehyde hydrolase | *dmpD (xylF)* | EC 3.7.1.9 | K10216 |  |  |
| 2-hydroxymuconate-6-semialdehyde dehydrogenase | *xylG (praB)* | EC 1.2.1.85 | K10217 |  |  |
| 2-hydroxymuconic semialdehyde dehydrogenase | *dmpC* | EC 1.2.1.32 |  |  |  |
| 4-oxalocrotonate tautomerase / 4-oxalocrotonate isomerase | *xylH (dmpI)* | EC 5.3.2.6 | K01821 |  |  |
| 4-oxalocrotonate decarboxylase | *dmpH (mhpD)* | EC 4.2.1.80 | K02554 |  |  |
| 2-oxopent-4-enoate hydratase | *dmpE (xylJ)* | EC 4.2.1.80 | K18364 |  |  |
| 4-hydroxy 2-oxovalerate aldolase | *dmpG (mhpE)* | EC 4.1.3.39 | K01666 |  |  |
| acetaldehyde dehydrogenase | *dmpF (mhpF)* | EC 1.2.1.10 | K04073 |  |  |
| S-(hydroxymethyl)glutathione dehydrogenase / alcohol dehydrogenase | *frmA*  *(ADH5)* | EC 1.1.1.284 / 1.1.1.1 | K00121 | Metabolism of xenobiotics by cytochrome p450 | |
| glutathione S-transferase | *gst* | EC 2.5.1.18 | K00799 |  |  |
| alcohol dehydrogenase, propanol-preferring | *adhP* | EC 1.1.1.1 | K13953 |  |  |
| Ammonia monooxygenase | *amoA* | EC 1.14.99.39 | K10944 | Flavin-dependent  monooxygenase | |
| Nitrilotriacetate monooxygenase component B flavin reductase) | *hpaC* | EC 1.5.1.36 | K00484 |  |  |
| Ubiquinone biosynthesis monooxygenase UbiB | *Coq7* | EC 1.14.13.- | K06134 |  |  |
| Alkanesulfonate monooxygenase | *ssuD* | EC 1.14.14.5 | K04091 |  |  |
| -4-hydroxyacetophenone monooxygenase | *HapE (pamO)* | EC 1.14.13.84 | K14520 |  |  |
| Toluene-4-monooxygenase system protein A | *tmoA* | EC 1.14.13.236 / 1.14.13.- | K15760 |  |  |
| Alkanal monooxygenase alpha chain | *luxA* | EC 1.14.14.3 | K00494 |  |  |
| Monooxygenase, flavin-binding family | *ChnB*  (cyclohexanone monooxygenase*)* | EC 1.14.13.22 | K03379 |  |  |
|  | *EthA*  *(monooxygenase)* | EC 1.14.13.- | K10215 |  |  |
| Dodecin Flavin-binding | *...* | ... | K09165 | … | |
| Amine oxidase, flavin-containing | *...* | ... | ... | ... | |
| Amine oxidase, flavin-containing | *...* | ... | ... | ... | |
| NADH:flavin oxidoreductases, Old Yellow Enzyme family | *fadH* | EC 1.3.1.34 | K00219 | ... | |

**Table S11** Gene annotations of *P.* *pseudoalcaligenes* strain *YKJ* genome related to its secretion system

| Definition | KEGG  Gene Name | EC  number | Ko  number | Type | KEGG Pathway |
| --- | --- | --- | --- | --- | --- |
| general secretion pathway protein D | *gspD* | ... | K02453 | II | Bacterial secretion system |
| general secretion pathway protein S | *GspS* | ... | K02465 |  |  |
| general secretion pathway protein O | *GspO* | EC 3.4.23.43 / 2.1.1.- | K02464 |  |  |
| general secretion pathway protein H | *GspH* | ... | K02457 |  |  |
| general secretion pathway protein C | *GspC* | ... | K02452 |  |  |
| general secretion pathway protein E (ATPase) | *gspE* | ... | K02454 |  |  |
| general secretion pathway protein F | *gspF* | ... | K02455 |  |  |
| general secretion pathway protein G | *gspG* | ... | K02456 |  |  |
| general secretion pathway protein I | *gspI* | ... | K02458 |  |  |
| general secretion pathway protein J | *gspJ* | ... | K02459 |  |  |
| general secretion pathway protein K | *gspK* | ... | K02460 |  |  |
| general secretion pathway protein L | *gspL* | … | K02461 |  |  |
| general secretion pathway protein M | *gspM* | ... | K02462 |  |  |
| YidC/Oxa1 family membrane protein insertase | *YidC* | ... | K03217 |  |  |
| preprotein translocase subunit SecA(ATPase) | *secA* | ... | K03070 | Sec-SRP |  |
| preprotein translocase subunit SecB | *SecB* | ... | K03071 |  |  |
| preprotein translocase subunit SecD | *secD* | ... | K03072 |  |  |
| preprotein translocase subunit SecE | *secE* | ... | K03073 |  |  |
| preprotein translocase subunit SecF | *secF* | ... | K03074 |  |  |
| preprotein translocase subunit SecY | *secY* | ... | K03076 |  |  |
| signal recognition particle subunit SRP54 | *ffh* | EC 3.6.5.4 | K03106 |  |  |
| fused signal recognition particle receptor | *ftsY* | ... | K03110 |  |  |
| preprotein translocase subunit YajC | *yajC* | ... | K03210 |  |  |
| sec-independent protein translocase protein TatA | *tatA* | … | K03116 | Tat |  |
| sec-independent protein translocase protein TatB | *tatB* | ... | K03117 |  |  |
| sec-independent protein translocase protein TatC | *tatC* | ... | K03118 |  |  |
| type IV secretion system protein VirD4 | *virD4* | ... | K03205 | IV |  |
| type IV secretion system protein VirB4 (ATPase) | *virB4* | ... | K03199 |  |  |
| type IV secretion system protein VirB11 (ATPase) | *virB11* | ... | K03196 |  |  |
| type IV secretion system protein VirB3 | *VirB3* | ... | K03198 |  |  |
| type IV secretion system protein VirB6 | *VirB6* | ... | K03201 |  |  |
| type IV secretion system protein VirB8 | *virB8* | ... | K03203 |  |  |
| type IV secretion system protein VirB10 | *VirB10* | ... | K03195 |  |  |
| type IV secretion system protein VirB1 | *VirB1* | ... | K03194 |  |  |
| type IV secretion system protein VirB2 | *virB2* | ... | K03197 |  |  |
| type IV secretion system protein VirB5 | *VirB5* | ... | K03200 |  |  |
| type IV secretion system protein VirB7 | *VirB7* | ... | K03202 |  |  |
| type IV secretion system protein VirB9 | *VirB9* | ... | K03204 |  |  |
| outer membrane protein | *tolC* | ... | K12340 | I |  |

**Table S12** Gene annotation of *P. pseudoalcaligenes* strain *YKJ* genome based on genes contributed in two component regulatory system

| Definition | KEGG Gene Name | EC number | Ko  number | KEGG Pathway |
| --- | --- | --- | --- | --- |
| cytochrome c oxidase cbb3-type subunit I | *ccoN* | EC 1.9.3.1 | K00404 | **Two-component system** |
| cytochrome c oxidase cbb3-type subunit II | *ccoO* | ... | K00405 |  |
| cytochrome c oxidase cbb3-type subunit III | *ccoP* | ... | K00406 |  |
| cytochrome c oxidase cbb3-type subunit IV | *ccoQ* | ... | K00407 |  |
| ubiquinol-cytochrome c reductase iron-sulfur subunit | *UQCRFS1 (petA)* | EC 1.10.2.2 | K00411 |  |
| ubiquinol-cytochrome c reductase cytochrome b subunit (petB) | *CYTB (petB)* | ... | K00412 |  |
| ubiquinol-cytochrome c reductase cytochrome c1 subunit | *CYC1 (petC)* | ... | K00413 |  |
| cytochrome d ubiquinol oxidase subunit I | *cydA* | EC 1.10.3.14 | K00425 |  |
| cytochrome d ubiquinol oxidase subunit II | *cydB* | EC 1.10.3.14 | K00426 |  |
| acetyl-CoA C-acetyltransferase | *atoB* | EC 2.3.1.9 | K00626 |  |
| [protein-PII] uridylyltransferase | *glnD* | EC.2.7.7.59 | K00990 |  |
| glutamine synthetase | *glnA* | EC 6.3.1.2 | K01915 |  |
| phosphate transport system substrate-binding protein | *pstS* | ... | K02040 |  |
| short-chain fatty acids transporter | *atoE* | ... | K02106 |  |
| cytochrome c oxidase assembly protein subunit 15 | *COX15* | ... | K02259 |  |
| chromosomal replication initiator protein | *dnaA* | ... | K02313 |  |
| negative regulator of flagellin synthesis FlgM | *flgM* | ... | K02398 |  |
| RNA polymerase sigma factor for flagellar operon FliA | *fliA* | ... | K02405 |  |
| flagellin | *fliC* | ... | K02406 |  |
| type IV pili sensor histidine kinase and response regulator | *pilL* | ... | K02487 |  |
| chemotaxis protein MotA | *motA* | ... | K02556 |  |
| twitching motility two-component system response regulator PilG | *pilG* | ... | K02657 |  |
| twitching motility two-component system response regulator PilH | *pilH* | ... | K02658 |  |
| twitching motility protein PilI | *pilI* | ... | K02659 |  |
| twitching motility protein PilJ | *pilJ* | ... | K02660 |  |
| two-component system, NtrC family, response regulator PilR | *pehR* | ... | K02667 |  |
| two-component system, NtrC family, sensor histidine kinase PilS | *pehS* | EC 2.7.13.3 | K02668 |  |
| RNA polymerase sigma-54 factor | *rpoN* | … | K03092 |  |
| methyl-accepting chemotaxis protein | *mcp* | ... | K03406 |  |
| two-component system, chemotaxis family, sensor kinase CheA | *cheA* | EC 2.7.13.3 | K03407 |  |
| purine-binding chemotaxis protein CheW | *cheW* | ... | K03408 |  |
| two-component system, chemotaxis family, protein-glutamate methylesterase/glutaminase | *cheB* | EC 3.1.1.6 /3.5.1.44 | K03412 |  |
| two-component system, chemotaxis family, chemotaxis protein CheY | *cheY* | ... | K03413 |  |
| two-component system, chemotaxis family, chemotaxis protein CheV | *cheV* | ... | K03415 |  |
| chemotaxis protein methyltransferase CheR | *cheR* | EC 2.1.1.80 | K00575 |  |
| carbon storage regulator | *csrA* | ... | K03563 |  |
| aerotaxis receptor | *aer* | ... | K03776 |  |
| serine protease Do | *htrA* | EC 3.4.21.107 | K04771 |  |
| chemosensory pili system protein ChpA (sensor histidine kinase/response regulator) | *chpA* | ... | K06596 |  |
| chemosensory pili system protein ChpB (putative protein-glutamate methylesterase) | *chpB* | ... | K06597 |  |
| chemosensory pili system protein ChpC | *chpC* | ... | K06598 |  |
| two-component system, OmpR family, phosphate regulon sensor histidine kinase PhoR | *phoR* | EC 2.7.13.3 | K07636 |  |
| two-component system, OmpR family, sensor histidine kinase PhoQ | *phoQ* | EC 2.7.13.3 | K07637 |  |
| two-component system, OmpR family, response regulator PhoP | *phoP* | ... | K07660 |  |
| two-component system, OmpR family, phosphate regulon response regulator PhoB | *phoB* | ... | K07657 |  |
| two-component system, OmpR family, osmolarity sensor histidine kinase EnvZ | *envZ* | EC 2.7.13.3 | K07638 |  |
| two-component system, OmpR family, sensor histidine kinase RstB | *rstB* | EC 2.7.13.3 | K07639 |  |
| two-component system, OmpR family, sensor histidine kinase CreC | *creC* | EC 2.7.13.3 | K07641 |  |
| two-component system, OmpR family, sensor histidine kinase QseC | *qseC* | EC 2.7.13.3 | K07645 |  |
| two-component system, OmpR family, sensor histidine kinase TctE | *tctE* | EC 2.7.13.3 | K07649 |  |
| two-component system, OmpR family, phosphate regulon response regulator OmpR | *ompR* | ... | K07659 |  |
| two-component system, OmpR family, response regulator RstA | *rstA* | ... | K07661 |  |
| two-component system, OmpR family, response regulator CpxR | *cpxR* | ... | K07662 |  |
| two-component system, OmpR family, response regulator QseB | *qseB* | ... | K07666 |  |
| two-component system, NarL family, nitrate/nitrite sensor histidine kinase NarX | *narX* | EC 2.7.13.3 | K07673 |  |
| two-component system, NarL family, sensor histidine kinase UhpB | *uhpB* | EC 2.7.13.3 | K07675 |  |
| two-component system, NarL family, sensor histidine kinase BarA | *barA* | EC 2.7.13.3 | K07678 |  |
| two-component system, NarL family, nitrate/nitrite response regulator NarL | *narL* | ... | K07684 |  |
| two-component system, NarL family, invasion response regulator UvrY | *uvrY* | ... | K07689 |  |
| two-component system, NtrC family, nitrogen regulation sensor histidine kinase GlnL | *GlnL (ntrB)* | EC 2.7.13.3 | K07708 |  |
| two-component system, NtrC family, nitrogen regulation response regulator GlnG | *glnG (ntrC)* | ... | K07712 |  |
| two-component system, OmpR family, response regulator TctD | *tctD* | ... | K07774 |  |
| Cu(I)/Ag(I) efflux system membrane protein CusA/SilA | *cusA* | ... | K07787 |  |
| multidrug efflux pump | *mdtB* | ... | K07788 |  |
| multidrug efflux pump | *mdtC* | ... | K07789 |  |
| putative tricarboxylic transport membrane protein | *tctA* | ... | K07793 |  |
| putative tricarboxylic transport membrane protein | *tctB* | ... | K07794 |  |
| putative tricarboxylic transport membrane protein | *tctC* | ... | K07795 |  |
| membrane fusion protein, Cu(I)/Ag(I) efflux system | *cusB* | ... | K07798 |  |
| membrane fusion protein, multidrug efflux system | *mdtA* | ... | K07799 |  |
| two-component system, LytTR family, sensor histidine kinase AlgZ | *algZ* | EC 2.7.13.3 | K08082 |  |
| two-component system, LytTR family, response regulator AlgR | *algR* | ... | K08083 |  |
| tetrathionate reductase subunit A | *ttrA* | ... | K08357 |  |
| tetrathionate reductase subunit B | *ttrB* | ... | K08358 |  |
| tetrathionate reductase subunit C | *ttrC* | ... | K08359 |  |
| two-component system, NtrC family, C4-dicarboxylate transport sensor histidine kinase DctB | *dctB* | EC 2.7.13.3 | K10125 |  |
| two-component system, NtrC family, C4-dicarboxylate transport response regulator DctD | *dctD* | ... | K10126 |  |
| CRP/FNR family transcriptional regulator, cyclic AMP receptor protein (clp) | *crp* | ... | K10914 |  |
| sigma-54 dependent transcriptional regulator, flagellar regulatory protein | *flrA* | ... | K10941 |  |
| two-component system, sensor histidine kinase FlrB | *flrB* | EC 2.7.13.3 | K10942 |  |
| two-component system, response regulator FlrC | *flrC* | ... | K10943 |  |
| C4-dicarboxylate-binding protein DctP | *dctP* | ... | K11688 |  |
| C4-dicarboxylate transporter, DctQ subunit | *dctQ* | ... | K11689 |  |
| C4-dicarboxylate transporter, DctM subunit | *dctM* | ... | K11690 |  |
| outer membrane protein | *tolC* | ... | K12340 |  |
| phosphatidylglycerol lysyltransferase | *mprF* | EC 2.3.2.3 | K14205 |  |
| two-component system, sensor histidine kinase RegB | *regB* | EC 2.7.13.3 | K15011 |  |
| two-component system, response regulator RegA | *regA* | ... | K15012 |  |
| two-component system, OmpR family, sensor histidine kinase PfeS | *pfeS* | EC 2.7.13.3 | K19609 |  |
| two-component system, sensor histidine kinase | *...* | EC 2.7.13.3 | K20975 |  |
| two-component system, HptB-dependent secretion and biofilm response regulator | *hsbR* | ... | K20977 |  |
| HptB-dependent secretion and biofilm anti anti-sigma factor | *hsbA* | ... | K20978 |  |
| putative two-component system response regulator | *...* | ... | K07814 |  |
| two-component system, OmpR family, catabolic regulation response regulator CreB | *creB* | ... | K07663 |  |

**Table S13** Gene annotation of *P. pseudoalcaligenes* strain *YKJ* genome based on genes associated with Quorum sensing process

| Definition | KEGG  Gene Name | EC  number | Ko  number | KEGG Pathway |
| --- | --- | --- | --- | --- |
| GTP cyclohydrolase II (ToxB) | *RibA* | EC 3.5.4.25 | K01497 | Quorum sensing system |
| 3-deoxy-7-phosphoheptulonate synthas (phzC) | *aroF* | EC 2.5.1.54 | K01626 |  |
| anthranilate synthase component I (phnA) | *trpE* | EC 4.1.3.27 | K01657 |  |
| anthranilate synthase component II  (phnB) | *trpG* | EC 4.1.3.27 | K01658 |  |
| long-chain acyl-CoA synthetase | *ACSL* | EC 6.2.1.3 | K01897 |  |
| branched-chain amino acid transport system ATP-binding protein | *livG* | ... | K01995 |  |
| branched-chain amino acid transport system ATP-binding protein | *livF* | ... | K01996 |  |
| branched-chain amino acid transport system permease protein | *livH* | ... | K01997 |  |
| branched-chain amino acid transport system permease protein | *livM* | ... | K01998 |  |
| branched-chain amino acid transport system substrate-binding protein | *livK* | ... | K01999 |  |
| putative spermidine/putrescine transport system ATP-binding protein | *ABC.SP.A* | ... | K02052 |  |
| putative spermidine/putrescine transport system permease protein | *ABC.SP.P* | ... | K02053 |  |
| putative spermidine/putrescine transport system permease protein | *ABC.SP.P1* | ... | K02054 |  |
| putative spermidine/putrescine transport system substrate-binding protein | *ABC.SP.S* | ... | K02055 |  |
| preprotein translocase subunit SecA | *secA* | ... | K03070 |  |
| preprotein translocase subunit SecB | *secB* | ... | K03071 |  |
| preprotein translocase subunit SecE | *secE* | ... | K03073 |  |
| preprotein translocase subunit SecY | *secY* | ... | K03076 |  |
| signal peptidase I | *lepB* | EC3.4.21.89 | K03100 |  |
| signal recognition particle subunit SRP54 | *SRP54* | EC 3.6.5.4 | K03106 |  |
| fused signal recognition particle receptor | *ftsY* | ... | K03110 |  |
| preprotein translocase subunit YajC | *yajC* | ... | K03210 |  |
| YidC/Oxa1 family membrane protein insertase | *yidC* | ... | K03217 |  |
| host factor-I protein | *hfq* | ... | K03666 |  |
| two-component system, OmpR family, sensor histidine kinase QseC | *qseC* | EC 2.7.13.3 | K07645 |  |
| two-component system, OmpR family, response regulator QseB | *qseB* | ... | K07666 |  |
| Fur family transcriptional regulator, zinc uptake regulator | *zur* | ... | K09823 |  |
| bacterial/archaeal transporter family-2 protein (ToxF) | *TC.BAT2* | ... | K09936 |  |
| CRP/FNR family transcriptional regulator, cyclic AMP receptor protein (clp) | *crp* | ... | K10914 |  |
| regulator of sigma E protease | *rseP* | EC 3.4.24.- | K11749 |  |
| diaminohydroxyphosphoribosylaminopyrimidine deaminase / 5-amino-6-(5-phosphoribosylamino)uracil reductase (ToxE) | *ribD* | EC 3.5.4.26 / 1.1.1.193 | K11752 |  |
| outer membrane protein, multidrug efflux system (ToxI) | *oprM* | ... | K18139 |  |
| type IV secretion system protein TrbJ | *TrbJ* | ... | K20266 |  |
| type IV secretion system protein TrbB | *trbB* | ... | K20527 |  |
| type IV secretion system protein TrbC | *trbC* | ... | K20528 |  |
| type IV secretion system protein TrbD | *trbD* | ... | K20529 |  |
| type IV secretion system protein TrbE | *trbE* | ... | K20530 |  |
| type IV secretion system protein TrbF | *trbF* | ... | K20531 |  |
| type IV secretion system protein TrbG | *trbG* | ... | K20532 |  |
| type IV secretion system protein TrbL | *trbL* | ... | K07344 |  |
| type IV secretion system protein TrbI | *trbI* | ... | K20533 |  |

**Table S14** Gene annotation of *P. pseudoalcaligenes* strain *YKJ* genome based on genes involved in antibiotic resistance pathways

| Definition | KEGG  Gene Name | EC  number | Ko  number | KEGG Pathway |
| --- | --- | --- | --- | --- |
| beta-N-acetylhexosaminidase | *nagZ* | EC 3.2.1.52 | K01207 | beta-Lactam resistance |
| membrane fusion protein, multidrug efflux system | *acrA* | ... | K03585 |  |
| cell division protein FtsI (penicillin-binding protein 3) | *ftsI* | EC 3.4.16.4 | K03587 |  |
| penicillin-binding protein 1A | *mrcA* | EC 2.4.1.129 / 3.4.16.4 | K05366 |  |
| penicillin-binding protein 2 | *mrdA* | EC 3.4.16.4 | K05515 |  |
| MFS transporter, PAT family, beta-lactamase induction signal transducer AmpG | *ampG* | ... | K08218 |  |
| Beta-lactamase class C and other penicillin binding proteins | *ampC* | EC 3.5.2.6 | K01467 |  |
| metallo-beta-lactamase family protein | *...* | ... | K07576 |  |
| Metal-dependent hydrolases of the beta-lactamase superfamily I; PhnP protein | *phnP* | EC 3.1.4.55 | K06167 |  |
| outer membrane protein | *tolC* | ... | K12340 |  |
| multidrug efflux pump | *acrB* | … | K18138 |  |
| outer membrane protein, multidrug efflux system (ToxI) | *oprM* | ... | K18139 |  |
| phospho-N-acetylmuramoyl-pentapeptide-transferase | *mraY* | EC 2.7.8.13 | K01000 | Vancomycin resistance |
| alanine racemase | *alr* | EC 5.1.1.1 | K01775 |  |
| D-alanine-D-alanine ligase | *ddl* | EC 6.3.2.4 | K01921 |  |
| UDP-N-acetylmuramoyl-tripeptide--D-alanyl-D-alanine ligase | *murF* | EC 6.3.2.10 | K01929 |  |
| UDP-N-acetylglucosamine--N-acetylmuramyl-(pentapeptide) pyrophosphoryl-undecaprenol N-acetylglucosamine transferase | *murG* | EC 2.4.1.227 | K02563 |  |
| chloramphenicol O-acetyltransferase type B | *catB* | EC 2.3.1.28 | K00638 | Cationic antimicrobial peptide (CAMP) |
| chloramphenicol-sensitive protein RarD | *rarD* | ... | K05786 |  |
| UDP-N-acetylglucosamine acyltransferase | *lpxA* | EC 2.3.1.129 | K00677 |  |
| N-acetylmuramoyl-L-alanine amidase | *amiA* | EC 3.5.1.28 | K01448 |  |
| membrane fusion protein, multidrug efflux system | *acrA* | ... | K03585 |  |
| thiol:disulfide interchange protein DsbA | *dsbA* | ... | K03673 |  |
| peptidyl-prolyl cis-trans isomerase A (cyclophilin A) | *PPIA* | EC 5.2.1.8 | K03767 |  |
| serine protease Do | *degP* | EC 3.4.21.107 | K04771 |  |
| two-component system, OmpR family, sensor histidine kinase PhoQ | *phoQ* | EC 2.7.13.3 | K07637 |  |
| two-component system, OmpR family, response regulator PhoP | *phoP* | ... | K07660 |  |
| two-component system, OmpR family, response regulator CpxR | *cpxR* | ... | K07662 |  |
| outer membrane protein | *tolC* | ... | K12340 |  |
| phosphatidylglycerol lysyltransferase | *mprF* | EC 2.3.2.3 | K14205 |  |
| multidrug efflux pump | *acrB* | ... | K18138 |  |

**Table S15** In Silico detection of *YKJ* plasmid DNA using PlasmidFinder server

| Enterobacteriaceae | | | | | |
| --- | --- | --- | --- | --- | --- |
| Plasmid | **%Identity** | **HSP Length/Query** | **Contig** | **Position in contig** | **Accession no.** |
| IncP1(Beta) | 99.14 | 583/582 | NODE_1_length_50798_cov_222.508 | 16484..17065 | U67194 |

**Table S16** the secretion system IV based on the *YKJ* plasmid DNA annotation

| **Category** | **Subcategory** | **Subsystem** | **Role** | **Features** |
| --- | --- | --- | --- | --- |
| Membrane Transport | Protein and nucleoprotein secretion system | Type IV | Conjugative transfer | Conjugative transfer protein TrbG |
|  |  |  |  | IncP-type DNA transfer protein TraD (Conjugative transfer protein TrbD) |
|  |  |  |  | Conjugative transfer protein TrbL |
|  |  |  |  | Conjugative transfer protein TrbF |
|  |  |  |  | Conjugative transfer protein TrbI |
|  |  |  |  | Conjugative transfer protein TrbJ |
|  |  |  |  | Conjugative transfer protein TrbB |
|  |  |  |  | IncP-type conjugative transfer protein TrbC |
|  |  |  |  | IncQ plasmid conjugative transfer protein TraQ (RP4 TrbM homolog) |
| DNA Metabolism | DNA replication | DNA topoisomerases | TypeI | ATP-independent DNA topoisomerase III (EC 5.99.1.2) |

**Table S17** Results of gene ontology studies based on *YKJ* plasmid DNA

| GO codes | activity | EC_number |
| --- | --- | --- |
| 0003917 | DNA topoisomerase type I | EC 5.99.1.2 |
| 0003676 | DNA primase (nucleic acid binding) | EC 2.7.7.- |
| 0008460 | dTDP-glucose 4,6-dehydratase | EC 4.2.1.46 |
| 0008831 | dTDP-4-dehydrorhamnose reductase | EC 1.1.1.133 |
| 0008879 | glucose-1-phosphate thymidylyltransferase | EC 2.7.7.24 |
| 0008830 | dTDP-4-dehydrorhamnose 3,5-epimeras | EC 5.1.3.13 |
| 0008846 | serine-type endopeptidase | - |
| 0006814 | sodium ion transport | - |
| 0008508 | bile acid:sodium symporter | - |
| 0016020 | Undecaprenyl-phosphate N-acetylglucosaminyl 1-phosphate transferase | EC 2.7.8.- |
|  | Arsenical-resistance protein ACR3 |  |
| 0008794 | arsenate reductase (glutaredoxin) | EC 1.20.4.1 |
| 0004365 | glyceraldehyde-3-phosphate dehydrogenase (NAD+) (phosphorylating) | EC 1.2.1.12 |
| 0008888 | glycerol dehydrogenase [NAD+] | EC 1.1.1.6 |
| 0003697 | single-stranded DNA binding |  |
| 0004316 | 3-oxoacyl-[acyl-carrier-protein] reductase (NADPH) | EC 1.1.1.100 |
| 0003697 | single-stranded DNA binding | - |
| 0003917 | DNA topoisomerase type I | EC 5.99.1.2 |
| 0003676 | nucleic acid binding | - |
| 0003677 | DNA binding | - |
| 0003896 | DNA primase | EC 2.7.7.- |
| 0006260 | DNA replication | - |
| 0006304 | DNA modification | - |
| 0008270 | zinc ion binding | - |
| 0003677 | DNA binding | - |
| 0003684 | damaged DNA binding | - |
| 0005524 | ATP binding | - |
| 0006259 | DNA metabolic process | - |
| 0006281 | DNA repair | - |
| 0006298 | DNA mismatch repair protein MutS | - |
| 0018836 | alkylmercury lyase | EC 4.99.1.2 |
| 0003700 | transcription factor activity, sequence-specific DNA binding | - |
| 0005622 | Arsenical resistance operon repressor (intracellular ) | - |
| 0003978 | UDP-glucose 4-epimerase | EC 5.1.3.2 |
| 0003824 | Undecaprenyl-phosphate N-acetylglucosaminyl 1-phosphate transferase (catalytic Activity ) | EC 2.7.8.- |
| 0006629 | lipid metabolic process | - |
| 0008761 | UDP-N-acetylglucosamine 2-epimerase | EC 5.1.3.14 |
| 0003677 | DNA binding | EC 2.7.7.- |
| 0004798 | thymidylate kinase | EC 2.7.4.9 |
| 0005525 | GTP-binding protein TypA/BipA (translation) |  |
| 0006412 | GTP-binding protein TypA/BipA (translation) | - |
| 0003979 | UDP-glucose 6-dehydrogenase | EC 1.1.1.22 |
| 0008761 | UDP-N-acetylglucosamine 2-epimerase | EC 5.1.3.14 |

**Table S18** The aromatic compound concentrations which strain *YKJ* showed maximum growth in the presence of them

| amount | aromatic compound |
| --- | --- |
| 1 (mM) | Phenol |
| 0.5 (g/l) | Benzoic acid |
| (v/v)0.1% | Benzene |
| (v/v) 0.1% | Chlorobenzene |
| (v/v)0.1% | Xylene |
| (v/v)0.1% | Styrene |
| 0.3(g/l) | Bisphenol A |

**Table S19** The investigation results of antibiotics resistance in *YKJ* strain

| Bacterial Growth | Antibiotic Concentrations (µg) | Antibiotic Names |
| --- | --- | --- |
| + | 100 | Ampicillin |
| - | 10-12/5 | Tetracycline |
| - | 50 | Kanamycin |
| - | 100 | Rifampicin |
| +++ | 25 | Chloramphenicol |

(The + sign indicates the intensity of bacterial growth *)
